# Elevated CO_2_ Improves the Physiology but Not the Final Yield in Spring Wheat Genotypes Subjected to Heat and Drought Stress During Anthesis

**DOI:** 10.3389/fpls.2022.824476

**Published:** 2022-03-07

**Authors:** Lamis Osama Anwar Abdelhakim, Thayna Mendanha, Carolina Falcato Fialho Palma, Ondřej Vrobel, Nikola Štefelová, Sanja Ćavar Zeljković, Petr Tarkowski, Nuria De Diego, Bernd Wollenweber, Eva Rosenqvist, Carl-Otto Ottosen

**Affiliations:** ^1^Department of Food Science, Plant, Food and Climate, Aarhus University, Aarhus, Denmark; ^2^Centre of Region Haná for Biotechnological and Agricultural Research, Czech Advanced Technology and Research Institute, Palacký University, Olomouc, Czechia; ^3^Department of Genetic Resources for Vegetables, Centre of the Region Haná for Biotechnological and Agricultural Research, Medicinal and Special Plants, Crop Research Institute, Olomouc, Czechia; ^4^Department of Agroecology, Crop Health, Aarhus University, Slagelse, Denmark; ^5^Department of Plant and Environmental Sciences, Crop Sciences, University of Copenhagen, Taastrup, Denmark

**Keywords:** wheat, elevated CO_2_, heat stress, gas exchange, chlorophyll fluorescence, targeted metabolomic analysis, grain yield

## Abstract

Heat and drought events often occur concurrently as a consequence of climate change and have a severe impact on crop growth and yield. Besides, the accumulative increase in the atmospheric CO_2_ level is expected to be doubled by the end of this century. It is essential to understand the consequences of climate change combined with the CO_2_ levels on relevant crops such as wheat. This study evaluated the physiology and metabolite changes and grain yield in heat-sensitive (SF29) and heat-tolerant (LM20) wheat genotypes under individual heat stress or combined with drought applied during anthesis at ambient (aCO_2_) and elevated CO_2_ (eCO_2_) levels. Both genotypes enhanced similarly the WUE under combined stresses at eCO_2_. However, this increase was due to different stress responses, whereas eCO_2_ improved the tolerance in heat-sensitive SF29 by enhancing the gas exchange parameters, and the accumulation of compatible solutes included glucose, fructose, β-alanine, and GABA to keep water balance; the heat-tolerant LM20 improved the accumulation of phosphate and sulfate and reduced the lysine metabolism and other metabolites including N-acetylornithine. These changes did not help the plants to improve the final yield under combined stresses at eCO_2_. Under non-stress conditions, eCO_2_ improved the yield of both genotypes. However, the response differed among genotypes, most probably as a consequence of the eCO_2_-induced changes in glucose and fructose at anthesis. Whereas the less-productive genotype LM20 reduced the glucose and fructose and increased the grain dimension as the effect of the eCO_2_ application, the most productive genotype SF29 increased the two carbohydrate contents and ended with higher weight in the spikes. Altogether, these findings showed that the eCO_2_ improves the tolerance to combined heat and drought stress but not the yield in spring wheat under stress conditions through different mechanisms. However, under non-stress conditions, it could improve mainly the yield to the less-productive genotypes. Altogether, the results demonstrated that more studies focused on the combination of abiotic stress are needed to understand better the spring wheat responses that help the identification of genotypes more resilient and productive under these conditions for future climate conditions.

## Introduction

Plants have to respond and adapt to multiple environmental changes, whose frequency is due to climate variability (Calanca, 2017). Heat and drought stresses are some of the major environmental stress factors limiting crop productivity. The combination of heat and drought causes severe impacts on physiological traits that affect plant growth and yield (Suzuki et al., 2014). Consequently, to sustain the future productivity for major crops such as wheat (*Triticum aestivum* L.), it is crucial to select tolerant cultivars that can ensure yield under combined abiotic stresses such as heat and drought (Zandalinas et al., 2018). Besides, global warming is expected to be accompanied by an increase in the atmospheric CO_2_ concentration that is expected to exceed 450 ppm by 2030 and to be above 720 ppm by 2100 (IPCC, 2007, 2014; Meehl et al., 2007). Thus, in the following years, plants will need to deal with the combination of heat and drought events in parallel with the increase of atmospheric CO_2_ concentration.

Plants grown under high temperatures reduce photosynthetic rates due to the limitation in the electron transport rate, reductions in the maximum efficiency of photosystem II (PSII), and downregulation of PSII photochemistry (Fahad et al., 2017). In addition, the lower affinity of Rubisco for CO_2_ fixation and the increase of the enzyme catabolism may also limit the increase in photosynthesis (Salvucci and Crafts-Brandner, 2004). However, this response differs among genotypes (Sharma et al., 2015). Whereas the heat-tolerant cultivars maintain the transpiration and, therefore, the photosynthesis at high temperatures as an adaptive mechanism under well-watered conditions, the heat-sensitive cultivars close stomata and consequently increase the leaf temperatures (Sharma et al., 2015).

At constant light levels and CO_2_ concentrations, water availability and temperature are the major drivers of plant stress responses (Will et al., 2013). The plants responded differently to the combination of different stresses than an individual stress type (Mittler, 2006; Suzuki et al., 2014). Heat stress, in combination with drought, causes alterations in plant growth and development (Suzuki et al., 2014). The detrimental impact on plants under the combined abiotic stresses depends on the stress severity, resulting in different regulating mechanisms (Suzuki et al., 2014). Moreover, the effect of higher temperatures on plant development and grain formation depends on the stress intensity and the developmental stage of the plant when the stress events occur (Farooq et al., 2011; Akter and Islam, 2017). For example, the combination of heat and drought stress during anthesis impairs fertilization of spikes and thereby reduces grain yield (Prasad et al., 2015). Heat and drought stresses also affect physiological traits, including photosynthesis, and limit biomass and crop productivity in wheat (Hlaváčová et al., 2018) and barley (Rollins et al., 2013). Thus, on the grain filling stage, they reduce the enzymatic activity in the starch and sucrose metabolism and, hence, the synthesis and translocation of photoassimilates from leaves to the reproductive organs (Sehgal et al., 2018). Opposite effects occur regarding stomatal regulation under the combination of heat and drought stress. Under these conditions, plants try to find a balance between avoiding the overheating of leaves by increasing transpiration and preventing water loss by decreasing the stomatal conductance (Prasch and Sonnewald, 2015). The stomata regulation is affected by the changes in temperature and humidity, indicating that higher temperatures may intensify the effects of drought (Will et al., 2013).

To mitigate the adverse effects induced by the combined stresses, plants activate metabolic changes as an acclimation mechanism (Suzuki et al., 2014). Among them, the changes of primary metabolites, such as carbohydrates, organic acids, and free amino acids, and secondary metabolites like polyamines and phenolic compounds play an essential role in regulating plant abiotic stress tolerance (Kaplan et al., 2004; Khan et al., 2020). Therefore, metabolic profiling of the plants under different growth conditions is essential to determine the metabolites implicated in the plant adaptation mechanisms (Kaplan et al., 2004; Khan et al., 2020). For example, carbohydrates are involved in osmotic adjustment and regulate membrane stability under abiotic stress (Farooq et al., 2009; Saddhe et al., 2021). The accumulation of certain organic acids such as malate enhances drought tolerance because of its role in maintaining the redox equilibrium between cell compartments, supporting ionic gradients on membranes, or regulating stomatal movement (Igamberdiev and Eprintsev, 2016; Marček et al., 2019). The accumulation of amino acids improves plant tolerance when grown under heat (Wang et al., 2018) or drought stress (De Diego et al., 2013). In the latest, the authors pointed to the free amino acids proline (Pro), glutamic acid (Glu), γ-aminobutyric acid (GABA), and the free polyamine spermine (Spm) as the main metabolites responsible for plant stress tolerance. Similarly, the accumulation of phenolic compounds improves plant tolerance against environmental stresses by reducing the oxidative damage of reactive oxygen species (ROS) as an antioxidant defense mechanism (Caverzan et al., 2016; Šamec et al., 2021).

The impact of elevated CO_2_ (eCO_2_) on the plant physiological responses has been also studied in wheat in combination with drought (Xu et al., 2016; Li et al., 2017) or heat stress (Shanmugam et al., 2013; Chavan et al., 2019), individually. However, there are a limited number of studies combining these two stressors in combination with eCO_2_ in wheat (Li et al., 2019; Abdelhakim et al., 2021). In summary of these aforementioned studies, the authors showed that eCO_2_ could alleviate the negative effect of drought by regulating the stomatal closure, lowering both stomatal conductance and transpiration rate, increasing water use efficiency (WUE), and thereby improving water status and plant growth (Xu et al., 2016; Li et al., 2017). When plants are exposed to high temperatures, eCO_2_ also improved plant performance by mitigating the photochemical damage of the plant due to a better electron transport rate as a result of higher Rubisco carboxylation efficiency and lower photorespiration (Pan et al., 2018; Chavan et al., 2019). The better photosynthetic efficiency contributes to higher biomass and yield production of wheat if no limitations have the prevailing effect (Parry et al., 2011). However, the response to eCO_2_ is suggested to be genotype dependent in many plant species including wheat (Tausz et al., 2013; Tausz-Posch et al., 2015). Further studies are needed to understand not only the acclimation mechanisms in different genotypes of wheat based on their heat susceptibility under combined heat and drought stress but also together with different CO_2_ scenarios.

It is clear that, to address the complex response of plants to multiple stress events, more studies should determine the physiological and metabolic mechanisms of plants (Mir et al., 2012). For that, this study aimed to investigate the physiological and metabolic responses in two spring wheat genotypes that differ in the response to heat stress during anthesis under combined heat stress and drought at ambient CO_2_ (aCO_2_) and eCO_2_. Additionally, the genotypes were brought to production to study the effect of combined stressors on the final yield and to evaluate the possible mitigation effect of eCO_2_. We hypothesized that the effect of eCO_2_ on plant stress tolerance and final yield would be genotype dependent and different for each plant growth condition.

## Materials and Methods

### Plant Material and Growing Conditions

In this study, 24 Nordic spring wheat (*Triticum aestivum* L.) genotypes were subjected to heat stress (at 40°C for 3 days) followed by recovery for 7 days in a climate chamber as a preliminary heat screening experiment to select the genotypes and classify them as heat sensitive and tolerant according to lower and higher maximum quantum efficiency of PSII photochemistry (F_*v*_/F_*m*_) values (Supplementary Table 1). Based on previous heat screening studies on wheat, it was reported that F_*v*_/F_*m*_ is a vital parameter used as a stress indicator under high temperatures to detect the heat susceptibilities in different genotypes (Baker and Rosenqvist, 2004; Sharma et al., 2012). The analysis ended with the classification of the genotypes in two categories: tolerant and sensitive. Among them, two spring wheat genotypes with high germination capacity were selected: one represents the heat-sensitive [SF29 (Sejet Plant Breeding, Denmark)] with F_*v*_/F_*m*_ of 0.562 ± 0.038, and another one represents the heat-tolerant [LM20 (Lantmännen, Sweden)] with F_*v*_/F_*m*_ of 0.707 ± 0.013. The main experiment was conducted under controlled conditions; seeds from both genotypes were sown in plastic pots (a 19-cm diameter, a 17-cm height, and a 3.1-L capacity) filled with a commercial peat substrate (Pindstrup Faerdigblanding 2, Pindstrup Mosebrug A/S, Ryomgaard, Denmark). Pots were divided into two groups, and each group was placed into independent compartments inside the greenhouse at the Department of Food Science, Aarhus University, Denmark: one with ambient CO_2_(aCO_2_) set at 400 ppm and another one with elevated CO_2_(eCO_2_) set at 800 ppm. The growth conditions in the greenhouse were set to long photoperiod conditions (16-h/8-h day/night) with natural light, which was supplemented by LED lamps (FL300 Grow, Senmatic, Søndersø, Denmark) when daytime photosynthetic photon flux density (PPFD) was below 150 μmol m^–2^ s^–1^. A dataTaker (DT605, Thermo Fisher Scientific, Australia), with a thermometer (4-wire PT100, RS Pro, GB), a humidity sensor (HMP60, Vaisala, Finland), and a quantum sensor (Li-Cor, United Kingdom) recorded climate data in the greenhouse. A climate control (LCC4, Senmatic A/S, Søndersø, Denmark) regulated the climate parameters inside the compartments. During the experiment, the aCO_2_ compartment maintained a temperature of 25 ± 2/17 ± 1°C day/night, relative humidity (RH) of 62 ± 8%, an average PPFD of 404 ± 85 μmol m^–2^ s^–1^, and CO_2_ concentration of 462 ± 43 ppm. The eCO_2_ compartment maintained a temperature of 25 ± 2/17 ± 1°C day/night, an RH of 64 ± 8%, an average PPFD of 354 ± 105 μmol m^–2^ s^–1^ and CO_2_ concentration of 771 ± 89 ppm. All the plants were fertigated by flooding on the greenhouse irrigation benches with a nutrient solution mix (191 ppm N; 27 ppm P; 171 ppm K, 20 ppm Mg, 170 ppm Ca, pH: 5.8, EC: 1.99 mS m^–1^) and supported by adding a support net (TEKU, STG19).

Once the plants reached the anthesis phase (Zadoks 61–65), at least five plants as a biological replicate per genotype and treatment were transferred into two different climate chambers (a PhytoScope FS-WI walk-in growth chamber, PSI, Czech Republic). The climate chamber conditions under control were set to mimic the greenhouse conditions. The RH was programmed to 60% and the CO_2_ concentration into two different levels: either 400 or 800 ppm. The light regime was also 16-h/8-h day/light, switching on at 6:00 PPFD of 150 μmol m^–2^ s^–1^ for 1 h, and then increasing to PPFD of 300 μmol m^–2^s^–1^ and maintaining this intensity until 21:00, from which the PPFD was reduced to 150 μmol m^–2^ s^–1^ for 1 h. After that, the light was switched off. The climate chambers were used in randomized order, depending on the sequence of the treatments. Besides, swapping the climate conditions in the climate chambers was also conducted with additional three biological replicates per genotype and treatment, ending with a total of eight plants as a biological replicate per variant.

### Application of Single and Combined Stresses

Before starting the treatments, the plants were acclimatized to the control for 2 days into the climate chambers, and the physiological status of the plants was measured before the stress treatments. After acclimation, the plants were subjected to different growth conditions, including (i) control (C), (ii) heat stress with irrigation (H), and (iii) a combination of drought and heat stress (D + H). In H and D + H treatments, the day started at 6:00 with a temperature of 32°C for 1 h, and, after that, it was increased to 36°C and maintained until 21:00, when the temperature was reduced to 32°C for 1 h. Finally, the temperature was set up to 28°C for the night regime. The heat stress was applied for 7 days; the first 4 days, the treated plants were subjected only to heat stress (H4). After that, the plants were divided into two groups: irrigated (H7) and non-irrigated (D + H7) plants and maintained for additional 3 days (a total of 7 days), ending with 8 biological replicates for each variant. The irrigation protocol was controlled through drought spotters (Phenospex, Heerlen, The Netherlands), where each pot was placed on an individual weighing scale to maintain the soil relative water content (SRWC) above 85% for the control plants. The D + H7-treated plants ended with an SRWC of 20%.

### Leaf Gas Exchange

Photosynthetic efficiency of the leaves per variant was determined using a portable gas exchange fluorescence system GFS-3000 (Walz, Effeltrich, Germany) with an integrated red/blue LED array and PAM-Fluorometer (3056-FL) to measure chlorophyll fluorescence parameters [e.g., operating efficiency of PSII (F_*q*_′/F_*m*_′) and electron transport rate (ETR)]. Both the photosynthetic CO_2_ and light response curves were conducted as performed by Abdelhakim et al. (2021). By fitting a model to these curves according to Sharkey et al. (2007), the fitted parameters from the photosynthetic CO_2_ response curves normalized at 25°C are the carboxylation rate by Rubisco (V_*c*,max_), photosynthetic electron transport (J_*max*_), and triose-phosphate utilization (TPU). Fitted parameters from the light response curves are the maximum net assimilation rate at light saturation (A_*max*_), the apparent quantum yield of CO_2_ assimilation (α), dark respiration rate (R_*dark*_), light compensation point (LCP), and convexity of the curve (θ) (Lobo et al., 2013). One flag leaf per plant was affixed in a 4-cm^2^ leaf cuvette. Six to eight biological replicates per variant during anthesis were measured. The cuvette temperature was set at 23°C for C and 36°C for H and D + H7, and the air-to-leaf vapor pressure deficit (VPD) was 11 ± 0.3 Pa kPa^–1^ at 23°C, and 23 ± 1 and 35.3 ± 1 Pa kPa^–1^ at 36°C under H and D + H7, respectively. Intrinsic water use efficiency (WUE_*i*_) and instantaneous WUE (WUE_*Leaf*_) were calculated from the ratios between the net photosynthetic rate (P_*n*_) and the stomatal conductance (g_*s*_) and transpiration rate (E), respectively, at PPFD of 2,000 μmol m^–2^ s^–1^.

### Chlorophyll Fluorescence

Chlorophyll fluorescence-related parameters of the wheat plants were measured using chlorophyll fluorometer PAM 2500 (Walz, Effeltrich, Germany) to assess photosynthetic performance under stress by assessing the changes in photosystem II (PSII) photochemistry in response to stress (Baker, 2008). The flag leaf of seven to eight plants per variant during anthesis was dark adapted for 25 min. Immediately after, according to the measurement of quenching analysis started first by turning on the measuring light to acquire a minimal level of fluorescence (F_*o*_), a saturation pulse is given to determine the maximal level of fluorescence (F_*m*_) and F_*v*_/F_*m*_ of an dark-adapted leaf, as described by Murchie and Lawson (2013). After a short dark relaxation of 40 s, actinic light was turned on at 1,500 μmol m^–2^ s^–1^ for 20 min to reach a steady state, and a sequence of 5 saturation pulses was applied at intervals to determine the non-photochemical quenching (NPQ) in a light-adapted state and the fraction of open PSII centers (oxidized Q_*A*_) (q_*L*_).

### Leaf Spectral Reflectance

Reflectance indices as photochemical reflectance index (PRI), derived from narrow-band reflectance at 531 and 570 nm and normalized difference vegetation index (NDVI), derived from near-infrared (reflected by a leaf) and red light (absorbed by a leaf) were determined by using a hand-held Poly-PlantPen RP 400 (PSI, Czech Republic). The measurements were taken on a light-adapted flag leaf of eight plants per variant during anthesis at the growing light condition (PPFD of 300 μmol m^–2^ s^–1^).

### Leaf Relative Water Content

At day 7 of stress, the flag leaf (ca., 4 cm) of eight plants per variant during anthesis was collected. The fresh weight (FW) of each sample was measured. Immediately after, they were soaked in distilled water overnight at room temperature. The sample was firstly wiped to remove the water excess and then weighed to determine the weight at full turgor (TW). Afterward, the samples were dried in an oven at 80°C for 24 h to measure the dry weight (DW). Leaf relative water content (LRWC) was calculated as follows: LRWC (%) = (FW-DW)/(TW-DW) × 100.

### Production and Grain Yield-Related Parameters

After the heat and combined stress treatments, the final yield was determined during anthesis (Z61), in which additional three wheat plants per variant were harvested, and the rest of the plants were placed back into the greenhouse until they reached full maturity during the ripening stage (Z92). At anthesis (Z61), the biomass was weighed; the number of leaves and tillers was counted. The leaf area was also measured using a leaf area meter (model 3100, LI-COR, Lincoln, Nebraska, United States) after the harvest. The samples were dried in an oven at 80°C for 72 h to determine dried total shoot biomass. In addition, these data allowed the calculation of the specific leaf area (SLA). During the ripening stage (Z92), the leaves, tillers, and spikes from eight plants per variant were also harvested and weighed individually. The spikes were then threshed to obtain the grain yield. Grain traits were determined through an optical measuring process by using a seed analyzer (MARViN ProLine, Germany). The seeds were filled in the seed tray for the optical measuring process, and then the seeds were weighed with a scale connected to the MARViN system. The MARViN software generated the grain parameters, including thousand-grain weight (TGW) and grain dimension (area, width, and length).

### Targeted Metabolomic Analysis

At day 7 of stress during the anthesis, the flag leaf from eight plants per variant was harvested, snap-frozen in liquid nitrogen, and stored at −80°C. The samples were lyophilized and the obtained DW was used for the targeted metabolomic analysis.

For the analysis of the free amino acids, pulverized plant material (3–5 mg) was mixed with 1 mL of 50% EtOH and sonicated for 10 min (Bandelin, Germany). After centrifugation (Prism, Labnet, United States) at 14,500 *g*, the supernatant was transferred into the new vial and kept at 20°C until analysis. For the quantification, 200 μL of supernatant was evaporated to dryness at 40°C under a vacuum (Centrivapm Labconco, United States), and then re-dissolved into 50 μL of the mobile phase, consisting from 20-mM ammonium formate, pH 3.0 (Component A), and 0.2% formic acid in ACN (Component B). UHPLC-MS/MS analysis was performed on Nexera X2 UHPLC (Shimadzu Handels GmbH), coupled with MS-8050 (Shimadzu Handels GmbH). Chromatographic separation was performed on an Acquity UPLC BEH AMIDE (50 × 2.1 mm; 1.7-μm particle size) with an appropriate pre-column. All target amino acids were separated using a binary gradient, starting at 90% B for 2 min, decreasing to 85% B for 2 min, and then decreasing to 65% B for 4 min, and then decreasing to 55% B for 1 min, and kept isocratic for 0.2 min. Initializing conditions were set after 0.1 min, and then equilibrating for 4.7 min. The column was kept at 40°C, and the flow rate was 0.4 mL min^−1^. The injection volume was 2 μL.

Free polyamines were analyzed according to the slightly modified method of Taibi et al. (2000). About 200 μL of 2-M NaOH was added into 200 μL of a supernatant, followed with 2.5 μL of benzoyl chloride (in MeOH, 50:50, v:v), and after vortexing for 5 s, the reaction mixture was stirred for 40 min at 25°C. About 500 μL of saturated NaCl was added, and benzoylated polyamines were extracted with 2 μL × 500 μL of diethyl ether. The solvent was evaporated under the vacuum at 40°C, and dry samples were dissolved in 200 μL of the mobile phase and analyzed according to the method described before by Marchetti et al. (2019).

The analysis of the three sugars: glucose (GLUC), fructose (FRUC), and sucrose (SUC) was performed according to the slightly modified method of O’Donoghue et al. (2004). Around 25 mg of lyophilized material was extracted with 1 mL of deionized water and filtered. The sugars were separated on a Rezex RCM monosaccharide Ca + column (300 mm × 7.8 mm, 8 μm). The detection was performed by ELSD under nitrogen flow of 2 L min^−1^ and a detector temperature of 80°C.

For quantifying the content of the phenolic compounds, homogenized plant material (10 mg) was mixed with 1 mL of 80% MeOH and sonicated for 10 min in an ultrasonic bath. After centrifugation at 14,500 *g*, the supernatant was transferred into the new vial and kept at −20°C until analysis. UHPLC-MS/MS analysis of free phenolic acids and flavonoids was performed according to the protocol described in our previous study (Zeljković et al., 2021).

Quantitative analyses of anions and organic acids were also carried out as previously described by Karalija et al. (2021). Commercially available two-part kit CElixirOA™ by MicroSolv (United States) was employed. Indirect UV detection is based on using 2,6-pyridinedicarboxylic acid as a background electrolyte (Soga and Ross, 1997). The extraction procedure was the same as for the quantitative measurement of saccharides. The identification of analytes was done by comparison with authentic standards provided by Sigma-Aldrich (Germany).

### Data Analysis

Three-way ANOVA was performed to determine the possible interaction between the three factors [genotypes (SF29 or LM20), treatment (C, H, or D + H) or CO_2_ levels (aCO_2_ or eCO_2_)]. Data were log-transformed to normalize them. Duncan’s test was used as a *post hoc* test for the multiple comparisons between the variants using SPSS 16.0 (SPSS Inc., Chicago, IL, United States). Multivariate statistical analysis was also carried out. Principal component analysis (PCA) was conducted using singular value decomposition, and PCA biplots were constructed. Hierarchical cluster analysis (HCA) was carried out, setting Euclidean distance as a similarity measure and complete linkage as the clustering method. Heatmaps with dendrograms were produced. Pearson correlations were computed and displayed. Three-way ANOVA, the *post hoc*, and the multivariate statistical analysis were performed in RStudio (R Software version 4.1.0), using packages *multicomp, agricolae, corrplot*, *ggplot2*, and *gplots*.

## Results

### Elevated CO_2_ Improved the CO_2_ Fixation but Not Fluorescence Parameters in Heat-Sensitive Spring Wheat Grown Under Heat Stress Combined With Drought

**FIGURE 1:**
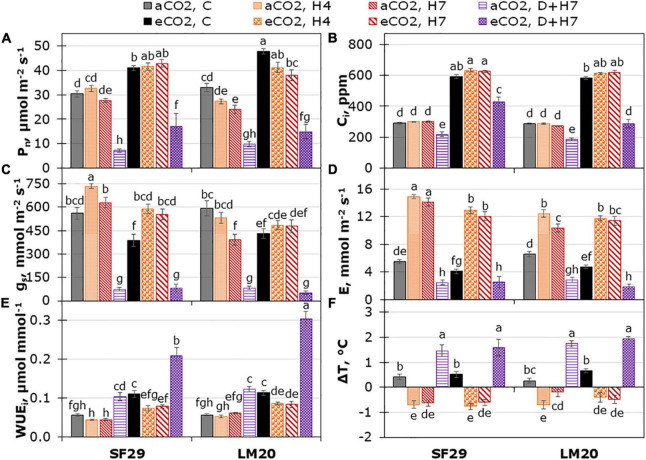
Leaf gas exchange measured at PPFD of 2,000 μmol m^– 2^ s^– 1^. **(A)** Net photosynthetic rate (P_*n*_), **(B)** intercellular CO_2_ (C_*i*_), **(C)** stomatal conductance (g_*s*_), **(D)** transpiration rate (E), **(E)** intrinsic water use efficiency (WUE_*i*_), and **(F)** difference between leaf and air cuvette temperature (ΔT) in heat-sensitive (SF29) and heat-tolerant (LM20) genotypes grown under control conditions (C), heat stress at Day 4 (H4), at Day 7 (H7), and combined heat stress and drought at Day 7 (D + H7) at ambient CO_2_ (aCO_2_) or elevated CO_2_ (eCO_2_). The data represent mean values ± standard error (S.E.) (*n* = 6–8). Different small letters indicate significant differences within variants according to Duncan’s test after ANOVA (*p* < 0.05).

As the first step to evaluate the effect of eCO_2_ in two spring wheat genotypes with different heat sensitivities grown under heat stress or the combination of heat stress and drought, we studied different physiological parameters related to the light and the dark phases of plant photosynthesis. Three-way ANOVA was performed to study the possible interaction between the three factors: genotype, treatment, and CO_2_ levels (Supplementary Table 2). The results showed that the parameters related to the dark phase of the photosynthesis such as P_*n*_, g_*s*_, C_*i*_, E, A_*max*_, and J_*max*_ were the most interesting because they were the only traits significantly affected by the interaction of the three factors, with *p*-values lower than 0.001 for all of them except for C_*i*_ (*p*-value = 0.009) and J_*max*_ (*p*-value = 0.014). The data were obtained from the light response curves conducted in both wheat genotypes grown under control conditions or heat stress alone or in combination with drought and at different CO_2_ levels (Supplementary Figures 1, 2), in which the parameters were discriminated at PPFD of 2,000 μmol m^–2^ s^–1^. As presented in Figure 1, eCO_2_ improved the P_*n*_ and C_*i*_ in both genotypes under all treatments, especially in the sensitive genotype SF29. Contrarily, both genotypes reduced the g_*s*_ and E under control conditions at eCO_2_ compared to aCO_2_ but did not change when they were grown under heat stress alone or combined with drought, except for SF29 that significantly reduced E under heat stress (Figures 1C,D).

**FIGURE 2:**
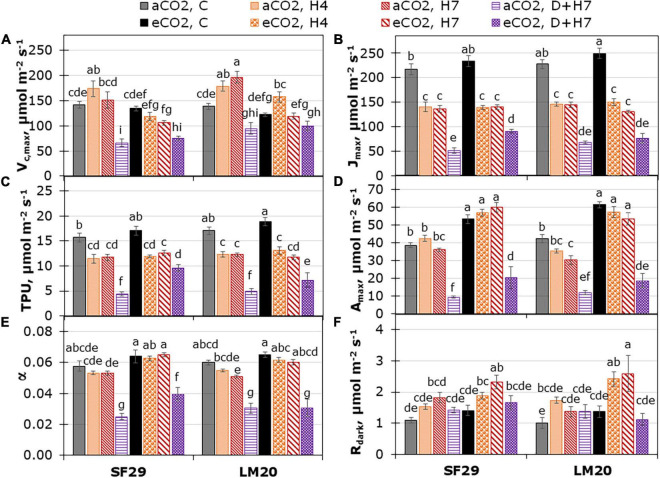
Fitted parameters normalized at 25°C from CO_2_ assimilation response curves; **(A)** Carboxylation rate by Rubisco (V_*c*,max_), **(B)** rate of photosynthetic electron transport (J_*max*_), **(C)** triose-phosphate utilization (TPU) and from light response curves; **(D)** maximum net CO_2_ assimilation rate at light saturation (A_*max*_), **(E)** apparent quantum yield of CO_2_ assimilation (α) and **(F)** dark respiration rate (R_*dark*_) in heat-sensitive (SF29) and heat-tolerant (LM20) genotypes grown under control conditions (C), heat stress at Day 4 (H4), at Day 7 (H7), and combined heat stress and drought at Day 7 (D + H7) at ambient CO_2_ (aCO_2_) or elevated CO_2_ (eCO_2_). The data represent mean values ± standard error (S.E.) (*n* = 6–8). Different small letters indicate significant differences within variants according to Duncan’s test after ANOVA (*p* < 0.05).

To understand more the different responses among genotypes, the WUE_*i*_ and WUE_*leaf*_ were also calculated (Figure 1E and Supplementary Figure 3A). In this case, we observed that there was no triple interaction, and the changes were due to the double interaction of the treatment with the CO_2_ levels, and also to the effect of the genotype in the case of WUE_*i*_ (*p*-values lower than 0.001) (Supplementary Table 2). In this case, we observed that WUE_*leaf*_ was significantly reduced in both genotypes under heat stress and combined with drought at aCO_2_, whereas WUE_*i*_ was not affected by the heat stress and increased under combined stresses (Figure 1E and Supplementary Figure 3A). This result pointed to WUE_*leaf*_ as a more sensitive parameter to stress than WUE_*i*_. However, the application of eCO_2_ significantly improved the WUE_*i*_ and WUE_*leaf*_ of both genotypes under all growth conditions compared to aCO_2_, except in the heat-tolerant LM20 under H7 for WUE_*i*_ (Figure 1E and Supplementary Figure 3A). Interestingly, the changes in the gas exchange and WUE parameters were not reflected in the ΔT, in which the changes were mainly due to the treatment effect, and there was no any influence of the CO_2_ levels (Figure 1F and Supplementary Table 2). Together with that, we calculated the LRWC of the plants and showed that the changes were mainly related to the treatment (Supplementary Figure 3B). Altogether, we could say that eCO_2_ helps the spring wheat to improve the gas exchange and WUE under all growth conditions, especially in the sensitive genotypes under combined stress, but this improvement is not affecting the effect of the stress in the temperature of the plants.

As a followed step, we analyzed the enzyme reaction rates associated with the dark reaction of photosynthesis. For that, the V_*c*,max_, J_*max*_, and TPU were estimated from the photosynthetic CO_2_ response curves normalized at 25°C (Figures 2A–C). As mentioned above, only J_*max*_ changed as a consequence of the interaction between the three factors: genotype, treatment, and CO_2_, whereas V_*c*,max_, and TPU presented double interaction between CO_2_ and treatment for both (*p*-value lower than 0.001), and between genotype and treatment for V_*c*,max_ (*P*-value = 0.005) (Supplementary Table 2). As a more relevant result, we observed that J_*max*_ was reduced in both genotypes as stress effect, being more affected under combined stress (D + H7) (Figure 2B). The application of eCO_2_ did not influence the stress-induced reduction of J_*max*_. Only the sensitive genotype SF29 improved this parameter under combined stress. This result could also explain better TPU in this genotype at the same growth conditions (Figure 2C).

Similar results were observed in the parameters obtained by fitting light response curves (Figures 2D–F), in which the A_*max*_ and α significantly increased only in SF29 when the plants were grown under combined stress (D + H7) at eCO_2_ compared to the same plants at aCO_2_ (Figures 2D,E). However, the eCO_2_ did not change the R_*dark*_, LCP, and θ, so the differences were mainly due to the plant growth conditions (Figure 2F, Supplementary Figures 4A,B, and Supplementary Table 2). Altogether, eCO_2_ induces a clear improvement of many parameters related to CO_2_ assimilation and water use efficiency, especially in the sensitive genotype when plants were grown under combined stresses.

**FIGURE 3:**
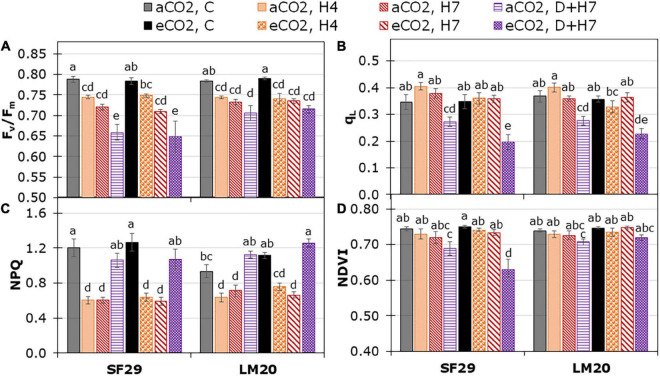
Chlorophyll fluorescence parameters; **(A)** the maximum quantum efficiency of PSII (F_*v*_/F_*m*_) on dark-adapted leaves, **(B)** fraction of open PSII centers (q_*L*_) and **(C)** non-photochemical quenching (NPQ), and the leaf reflectance represented as **(D)** normalized difference vegetation index (NDVI) in heat-sensitive (SF29) and heat-tolerant (LM20) genotypes grown under control conditions (C), heat stress at Day 4 (H4), at day D (H7), and combined heat stress and drought at Day 7 (D + H7) at ambient CO_2_ (aCO_2_) or elevated CO_2_ (eCO_2_). The data represent mean values ± standard error (S.E.) (*n* = 6–8). Different small letters indicate significant differences within variants according to Duncan’s test after ANOVA (*p* < 0.05).

The light phase of the plant photosynthesis was also evaluated by changes in the plant chlorophyll fluorescence, which was affected mainly by the treatment (*p*-values lower than 0.001); the single and combined stresses (Figures 3A–C, Supplementary Figure 2, and Supplementary Table 2). Only qL was affected by the different CO_2_ levels, with a significant reduction in SF29 plants when they were grown under combined stress (D + H7) at eCO_2_ compared to the plants at aCO_2_ (Figure 3B). These results could also explain the significant reduction in the vegetation index NDVI (related to plant greenness), where SF29 also reduced it under combined stresses at eCO_2_ (Figure 3D). However, no differences induced by the CO_2_ were observed in PRI (Supplementary Figure 4C). Altogether, eCO_2_ rather negatively affected the light phase of photosynthesis in the sensitive genotype grown under combined stresses (D + H7).

### The Elevated CO_2_ Activated Different Metabolic Strategies Among Genotypes Under Combined Heat Stress and Drought

To understand a bit more about the response of both genotypes under different growth conditions, the content of 46 metabolites, including carbohydrates, organic acids, phenolic compounds, free amino acids, and free polyamines, was quantified in the flag leaf harvested during anthesis (Table 1 and Supplementary Table 3). The analysis of these metabolites could give an idea of how the primary metabolism and the relationship between C and N are affected in both genotypes when they are grown under heat stress alone or in combination with drought at different CO_2_ levels. As the first step, we performed a three-way ANOVA for all metabolites and observed that several compounds changed due to the interaction between genotype, treatment, and CO_2_ (Table 1). Firstly, we analyzed the carbohydrates as direct photosynthesis products. We observed that glucose (GLUC) and fructose (FRUC) were significantly accumulated in both genotypes under heat stress alone and combined with drought at aCO_2_. The application of eCO_2_ increased the accumulation of both carbohydrates in SF29 plants under control conditions and heat stress but did not change under combined stresses compared to aCO_2_. The eCO_2_ also reduced the SUC content in SF29 under the detection levels. However, the eCO_2_ reduced the GLUC and FRUC levels in the heat-tolerant genotype LM20 under control conditions and combined stress (D + H7). GLUC and FRUC are used as carbon skeletons for the amino acid and organic acid synthesis through the Krebs cycles. For that, the levels of these compounds were also determined. No relevant changes were observed in organic acids, including Citr and Ma, intermediates of the Krebs cycle (Table 1). Only LM20 significantly increased the phosphate (PO) and sulfate (SO) content under D + H7 at eCO_2_ compared with aCO_2_ (Table 1). No relevant differences were observed regarding phenolic compounds. However, the genotypes changed the content of several free amino acids as a response to the growth conditions and CO_2_ levels (Table 1). For example, the heat-sensitive genotype SF29 significantly accumulated βAla, GABA, and Trp and reduced the levels of Cit, Orn, and Cyst when the plants were grown under combined stresses (D + H7) at eCO_2_ compared to at aCO_2_. However, LM20 tended to reduce Asn, Glu, His, HomoArg, and Lys, and, as a consequence, presented lower levels of the Lys catabolic product, the polyamine Cad, but without significant differences between the plants grown under D + H7 at eCO_2_ and aCO_2_ (Table 1). AcOrn was accumulated in both genotypes when they were grown under stress conditions (heat stress or combined with drought) at aCO_2_ and significantly reduced in LM20 under combined stresses at eCO_2_. However, this accumulation was reduced when the eCO_2_ was applied (Table 1). It is also worth mentioning that both genotypes significantly increased Pro levels when they were grown under combined stresses and being higher at eCO_2_, especially in the case of LM20 (Table 1).

**TABLE 1:** Primary and secondary metabolites in a heat-sensitive (SF29) and heat-tolerant (LM20) genotypes grown under control conditions (C), heat stress at day 7 (H7) and combined heat stress and drought at day 7 (D+H7) at ambient CO_2_ (aCO_2_) or elevated CO_2_ (eCO_2_).

Genotype		SF29						LM20					
Type	Metabolite	aCO_2_, C	aCO_2_, H7	aCO_2_, D+H7	eCO_2_, C	eCO_2_, H7	eCO_2_, D+H7	aCO_2_, C	aCO_2_, H7	aCO_2_, D+H7	eCO_2_, C	eCO_2_, H7	eCO_2_, D+H7
Sugars (nmol mg^–1^ DW)	FRUC	24.19 ± 1.63	44.4 ± 5.1	40.62 ± 1.47	71.75 ± 5.44	64.54 ± 4.57	37.46 ± 4.07	88.25 ± 10.22	68.96 ± 3.27	69.52 ± 4.84	36.87 ± 1.51	75.93 ± 3.73	39.3 ± 3.88
		**d**	**c**	**c**	**b**	**b**	**cd**	**a**	**b**	**b**	**cd**	**ab**	**c**
	GLUC	30.17 ± 1.52	56.92 ± 3.02	57.22 ± 3.98	90.22 ± 11.21	98.01 ± 6.87	45.49 ± 4.58	130.59 ± 11.68	94.21 ± 6.8	94.26 ± 6.75	33.42 ± 3.42	113.86 ± 7.28	45.13 ± 5.15
		**e**	**d**	**d**	**c**	**bc**	**de**	**a**	**bc**	**bc**	**e**	**ab**	**de**
	SUC	93.43 ± 7.29	74.25 ± 6.8	56.5 ± 4.98	*N* *D*	*N* *D*	*N* *D*	*N* *D*	*N* *D*	*N* *D*	*N* *D*	*N* *D*	*N* *D*
		**a**	**b**	**b**									
Organic acids (pmol mg^–1^ DW)	Cl	8.92 ± 1.33	9.21 ± 0.61	15.97 ± 1.25	11.21 ± 0.4	10.78 ± 0.74	13.88 ± 1.39	10.55 ± 0.47	9.04 ± 1.45	10.16 ± 1.01	10.69 ± 1.58	13.19 ± 0.8	14.58 ± 1.78
		**d**	**d**	**a**	**bcd**	**bcd**	**ab**	**cd**	**d**	**cd**	**bcd**	**abc**	**a**
	Citr	12.35 ± 1.24	9.59 ± 1.54	14.03 ± 1.85	12.75 ± 1.06	6.78 ± 1.07	10.14 ± 1.34	12.58 ± 1.13	8.89 ± 1.83	6.8 ± 0.73	11.46 ± 0.9	9.79 ± 0.92	8.78 ± 1.56
		**ab**	**bc**	**a**	**ab**	**c**	**abc**	**ab**	**bc**	**c**	**ab**	**bc**	**bc**
	Ma	4.16 ± 0.45	3.45 ± 0.55	5.12 ± 0.72	3.9 ± 0.25	2.74 ± 0.44	3.88 ± 0.45	6.94 ± 0.68	3.91 ± 0.49	4.66 ± 0.66	3.61 ± 0.25	3.03 ± 0.47	5.34 ± 0.93
		**bcde**	**cde**	**bc**	**bcde**	**e**	**bcde**	**a**	**bcde**	**bcd**	**bcde**	**de**	**b**
	NO3	5.93 ± 1.75	8.09 ± 1.36	21.51 ± 2.35	13.47 ± 4.19	8.95 ± 2.18	12.95 ± 3.24	2.75 ± 0.61	7.02 ± 0.82	16.9 ± 2.1	2.53 ± 0.87	4.87 ± 0.56	12.25 ± 2.03
		**de**	**cde**	**a**	**bc**	**cde**	**bc**	**e**	**cde**	**ab**	**e**	**e**	**bcd**
	PO	6.57 ± 0.52	4.88 ± 0.64	8.36 ± 1.22	8.32 ± 0.87	5.48 ± 0.46	7.82 ± 1.11	12.28 ± 1.08	7.05 ± 1.21	7.31 ± 1.09	8.65 ± 0.84	8.96 ± 0.87	11.66 ± 1.21
		**bcd**	**d**	**bc**	**bc**	**cd**	**bcd**	**a**	**bcd**	**bcd**	**b**	**b**	**a**
	SO	3.51 ± 0.64	5.54 ± 0.35	6.4 ± 0.82	4.82 ± 0.65	5.26 ± 0.49	7.85 ± 0.52	3.88 ± 0.42	3.74 ± 0.58	3.17 ± 0.39	1.37 ± 0.41	4.46 ± 0.75	5.26 ± 0.92
		**cd**	**bc**	**ab**	**bcd**	**bc**	**a**	**cd**	**cd**	**d**	**e**	**bcd**	**bc**
Free phenolics (pmol mg^–1^ DW)	4-HBA	0.047 ± 0.004	0.061 ± 0.01	0.086 ± 0.01	0.079 ± 0.02	0.154 ± 0.04	0.088 ± 0.01	0.056 ± 0.02	0.087 ± 0.02	0.075 ± 0.02	0.078 ± 0.03	0.122 ± 0.03	0.165 ± 0.03
		**d**	**cd**	**bcd**	**cd**	**ab**	**bcd**	**cd**	**bcd**	**cd**	**cd**	**abc**	**a**
	CHLA	0.609 ± 0.06	0.13 ± 0.04	0.035 ± 0.01	0.08 ± 0.03	0.051 ± 0.02	0.166 ± 0.09	0.026 ± 0.01	0.055 ± 0.02	0.013 ± 0	0.151 ± 0.09	0.02 ± 0.01	0.015 ± 0.01
		**a**	**b**	**b**	**b**	**b**	**b**	**b**	**b**	**b**	**b**	**b**	**b**
	FA	0.206 ± 0.01	19.037 ± 3.07	0.373 ± 0.08	0.321 ± 0.08	0.162 ± 0.03	0.422 ± 0.1	0.199 ± 0.03	0.206 ± 0.1	0.526 ± 0.09	0.28 ± 0.05	0.335 ± 0.1	0.183 ± 0.05
		**b**	**a**	**b**	**b**	**b**	**b**	**b**	**b**	**b**	**b**	**b**	**b**
	pCA	0.029 ± 0.004	0.043 ± 0.01	0.138 ± 0.07	0.023 ± 0.01	0.021 ± 0.002	0.045 ± 0.01	0.059 ± 0.01	0.026 ± 0.01	0.067 ± 0.01	0.043 ± 0.01	0.039 ± 0.01	0.047 ± 0.01
		**b**	**b**	**a**	**b**	**b**	**b**	**b**	**b**	**b**	**b**	**b**	**b**
	SaA	0.03 ± 0.01	0.376 ± 0.11	0.04 ± 0.01	0.111 ± 0.02	0.049 ± 0.01	0.024 ± 0.003	0.111 ± 0.02	0.043 ± 0.01	0.031 ± 0.01	0.04 ± 0.01	0.059 ± 0.01	0.032 ± 0.01
		**b**	**a**	**b**	**b**	**b**	**b**	**b**	**b**	**b**	**b**	**b**	**b**
	SaAG	1.684 ± 0.36	0.309 ± 0.06	0.52 ± 0.1	0.332 ± 0.04	0.353 ± 0.06	0.443 ± 0.09	4.796 ± 0.99	2.404 ± 0.34	3.927 ± 1.28	4.171 ± 0.83	2.082 ± 0.72	1.381 ± 0.32
		**cd**	**d**	**d**	**d**	**d**	**d**	**a**	**bc**	**ab**	**ab**	**cd**	**cd**
Free amino acids (pmol mg^–1^ DW)	AAA	0.04 ± 0.01	0.06 ± 0.01	4.14 ± 0.62	0.05 ± 0.01	0.06 ± 0.02	3.57 ± 0.46	0.11 ± 0.02	0.08 ± 0.02	6.45 ± 0.95	0.06 ± 0.01	0.07 ± 0.02	7.13 ± 0.68
		**c**	**c**	**b**	**c**	**c**	**b**	**c**	**c**	**a**	**c**	**c**	**a**
	AcGlu	2.1 ± 0.18	0.6 ± 0.08	0.16 ± 0.02	2.29 ± 0.4	1.05 ± 0.12	0.18 ± 0.01	2.89 ± 0.36	0.57 ± 0.11	0.21 ± 0.01	2.91 ± 0.27	0.71 ± 0.09	0.2 ± 0.03
		**b**	**cd**	**d**	**b**	**c**	**d**	**a**	**cd**	**d**	**a**	**cd**	**d**
	AcOrn	4.94 ± 0.87	85.8 ± 23.12	75.02 ± 12.32	3.45 ± 0.95	33.72 ± 6.36	45.19 ± 6.3	4.44 ± 0.89	25.49 ± 2.35	108.26 ± 24.61	8.89 ± 1.7	25.88 ± 3.42	68.12 ± 8.62
		**e**	**ab**	**bc**	**e**	**de**	**cd**	**e**	**de**	**a**	**e**	**de**	**bc**
	Ala	78.32 ± 13.63	190.01 ± 21.7	72.55 ± 11.77	86.95 ± 16.09	128.24 ± 16.66	69.05 ± 10.98	55.8 ± 3.93	73.01 ± 11.13	77.57 ± 10.74	66.43 ± 8.1	137.47 ± 22.72	42.5 ± 7.72
		**c**	**a**	**c**	**c**	**b**	**c**	**c**	**c**	**c**	**c**	**b**	**c**
	Arg	0.05 ± 0.02	0.1 ± 0.02	0.19 ± 0.06	0.06 ± 0.03	0.03 ± 0.01	0.23 ± 0.11	0.03 ± 0	0.03 ± 0.01	0.14 ± 0.03	0.02 ± 0.01	0.02 ± 0.01	0.07 ± 0.02
		**c**	**bc**	**ab**	**c**	**c**	**a**	**c**	**c**	**abc**	**c**	**c**	**bc**
	Asn	5.9 ± 1	85.99 ± 15.29	192.47 ± 34.82	8.82 ± 1.35	87.47 ± 21.1	201.89 ± 35.2	15.15 ± 1.68	28.19 ± 3.87	313.96 ± 60.24	31.29 ± 6.6	109.19 ± 23.79	223.1 ± 26.98
		**d**	**cd**	**b**	**d**	**cd**	**b**	**d**	**cd**	**a**	**cd**	**c**	**b**
	Asp	36.33 ± 5.68	110.4 ± 5.05	14.09 ± 3.41	21.3 ± 3.78	30.35 ± 7.18	26.64 ± 3.95	45.31 ± 8.21	36.19 ± 5.98	24.68 ± 4.44	58.17 ± 11.16	23.86 ± 4.05	15.48 ± 2.1
		**cd**	**a**	**e**	**de**	**cde**	**de**	**bc**	**cd**	**de**	**b**	**de**	**e**
	BABA	0.37 ± 0.06	0.27 ± 0.07	0.48 ± 0.1	0.57 ± 0.07	0.3 ± 0.06	0.48 ± 0.11	0.3 ± 0.03	0.28 ± 0.05	0.29 ± 0.07	0.42 ± 0.08	0.23 ± 0.02	0.26 ± 0.06
		**a**	**ab**	**ab**	**abc**	**abc**	**bc**	**bc**	**bc**	**bc**	**bc**	**bc**	**c**
	βAla	0.87 ± 0.19	1.16 ± 0.24	7.64 ± 1.48	0.64 ± 0.12	1.19 ± 0.22	11.83 ± 3.43	0.88 ± 0.14	0.61 ± 0.12	5.94 ± 0.7	1.17 ± 0.2	1.26 ± 0.17	7.03 ± 1.36
		**c**	**c**	**b**	**c**	**c**	**a**	**c**	**c**	**b**	**c**	**c**	**b**
	Cit	0.8 ± 0.16	0.57 ± 0.15	0.95 ± 0.37	0.42 ± 0.08	0.32 ± 0.06	0.34 ± 0.07	0.54 ± 0.04	0.32 ± 0.07	0.81 ± 0.2	1 ± 0.24	0.34 ± 0.08	0.62 ± 0.11
		**ab**	**ab**	**a**	**b**	**b**	**b**	**ab**	**b**	**ab**	**a**	**b**	**ab**
	Cyst	27.3 ± 1.04	14.01 ± 1.44	18.06 ± 2.07	15.7 ± 1.24	15.16 ± 0.8	10.79 ± 1.62	16.84 ± 1.47	12.47 ± 1.07	14.71 ± 1.23	20.87 ± 2.54	13.1 ± 0.61	11.04 ± 0.96
		**a**	**cde**	**bc**	**cd**	**cde**	**e**	**bcd**	**de**	**cde**	**b**	**de**	**e**
	GABA	9.53 ± 1.44	8.67 ± 1.36	9.19 ± 1.43	9.98 ± 1.49	6.67 ± 0.59	20.81 ± 3.63	10.67 ± 1.18	3.48 ± 0.52	9.79 ± 1.5	6.37 ± 0.85	6.47 ± 0.74	11.47 ± 1.36
		**b**	**b**	**b**	**b**	**bc**	**a**	**b**	**c**	**b**	**bc**	**bc**	**b**
	Gln	81.6 ± 11.75	157.61 ± 36.55	251.82 ± 39.24	65.34 ± 16.09	46.09 ± 8.11	197.32 ± 19.2	87.62 ± 10.47	93.05 ± 21.31	395.51 ± 46.02	80.06 ± 15.66	76.36 ± 14.27	328.89 ± 40.73
		**de**	**cd**	**b**	**e**	**e**	**bc**	**de**	**de**	**a**	**de**	**de**	**a**
	Glu	238.38 ± 31.92	109.01 ± 18.78	148.38 ± 17.1	163.48 ± 41.91	129.61 ± 17.04	108.11 ± 22.94	339.18 ± 41.22	188.17 ± 28.36	323.28 ± 53.61	283.45 ± 51.64	208.52 ± 36.51	185.2 ± 14.99
		**abc**	**d**	**cd**	**cd**	**cd**	**d**	**a**	**bcd**	**a**	**ab**	**bcd**	**bcd**
	His	2.4 ± 0.87	34.75 ± 7.38	20.47 ± 5.21	0.94 ± 0.15	11.63 ± 3.22	17.02 ± 1.04	1.31 ± 0.21	8.02 ± 0.81	48.37 ± 10.22	1.95 ± 0.44	5.21 ± 0.85	32.6 ± 4.8
		**e**	**b**	**c**	**e**	**cde**	**cd**	**e**	**cde**	**a**	**e**	**de**	**b**
	HomoArg	2.49 ± 0.47	49.11 ± 7.44	15.05 ± 3.57	1.77 ± 0.32	9.69 ± 2.78	11.34 ± 1.62	3.82 ± 0.83	6.46 ± 0.68	24.92 ± 5.38	3.55 ± 1.02	6.28 ± 0.9	13.16 ± 1.29
		**e**	**a**	**c**	**e**	**cde**	**cde**	**de**	**cde**	**b**	**de**	**cde**	**cd**
	Lys	1.38 ± 0.16	53.36 ± 10.6	34.63 ± 6.04	2.73 ± 0.5	35.82 ± 5.02	40.24 ± 7.59	2.38 ± 0.5	18.19 ± 2.45	172.08 ± 22.86	3.47 ± 0.82	10.76 ± 1.44	54.89 ± 9.62
		**e**	**b**	**bcd**	**e**	**bcd**	**bc**	**e**	**cde**	**a**	**e**	**de**	**b**
	Met	1.51 ± 0.23	3.51 ± 0.8	19.68 ± 3.48	1.74 ± 0.43	1.3 ± 0.2	16.49 ± 3.39	0.81 ± 0.1	1.09 ± 0.12	9.03 ± 0.93	0.55 ± 0.06	0.94 ± 0.11	11.43 ± 1.07
		**c**	**c**	**a**	**c**	**c**	**a**	**c**	**c**	**b**	**c**	**c**	**b**
	Orn	2.63 ± 0.25	3.12 ± 0.56	3.33 ± 0.66	1.67 ± 0.25	1.73 ± 0.25	1.62 ± 0.22	1.81 ± 0.18	2.01 ± 0.41	2.94 ± 0.3	1.9 ± 0.21	1.75 ± 0.26	2.9 ± 0.37
		**abcd**	**ab**	**a**	**d**	**d**	**d**	**cd**	**bcd**	**abc**	**cd**	**d**	**abc**
	Phe	5.5 ± 0.63	25.52 ± 3.08	123.19 ± 17.82	5.22 ± 0.67	4.77 ± 0.82	148.01 ± 20.67	4.66 ± 0.5	9.01 ± 1.66	116.63 ± 10.88	3.6 ± 0.61	16.5 ± 4.29	130.2 ± 7.35
		**c**	**c**	**ab**	**c**	**c**	**a**	**c**	**c**	**b**	**c**	**c**	**ab**
	Pro	9.55 ± 1.35	15.43 ± 2.7	202.3 ± 29.51	9.16 ± 2.24	6.71 ± 0.72	244.45 ± 53.15	18.85 ± 4.49	9.48 ± 1.68	236.86 ± 18.38	6.55 ± 0.47	9.82 ± 1.45	281.1 ± 15.3
		**c**	**c**	**b**	**c**	**c**	**ab**	**c**	**c**	**ab**	**c**	**c**	**a**
	Ser	112.28 ± 10.98	118.68 ± 19.11	131.98 ± 23.06	84.08 ± 12.98	81.08 ± 19.77	98.31 ± 11.78	160.66 ± 21.66	86.22 ± 20.66	150.65 ± 35.29	128.24 ± 21.07	85.68 ± 12.83	138.42 ± 23.44
		**abc**	**abc**	**abc**	**bc**	**c**	**abc**	**a**	**bc**	**ab**	**abc**	**bc**	**abc**
	Thr	65.14 ± 11.51	84.64 ± 14.32	96.54 ± 13.82	42.45 ± 6.59	59.08 ± 7.55	79.27 ± 10.23	49.21 ± 4.13	47.95 ± 9.38	97.76 ± 17.23	35.96 ± 4.72	67.23 ± 9.29	82.97 ± 7.95
		**abcd**	**ab**	**a**	**d**	**bcd**	**abc**	**cd**	**cd**	**a**	**d**	**abcd**	**ab**
	Trp	0.43 ± 0.12	14.87 ± 1.44	64.58 ± 10.19	2.16 ± 0.81	17 ± 3.56	100.73 ± 15.32	0.45 ± 0.08	15.71 ± 3.93	60.76 ± 7.48	0.42 ± 0.09	7.79 ± 1.23	75.78 ± 7.88
		**c**	**c**	**b**	**c**	**c**	**a**	**c**	**c**	**b**	**c**	**c**	**b**
	Tyr	9.19 ± 1.19	35.44 ± 5.96	135.42 ± 17.81	15.11 ± 4.6	24.46 ± 9.06	139.16 ± 23.53	8.89 ± 0.81	15.55 ± 2.33	120.07 ± 7.86	7.99 ± 0.66	15.56 ± 1.96	148.51 ± 9.84
		**b**	**b**	**a**	**b**	**b**	**a**	**b**	**b**	**a**	**b**	**b**	**a**
	Val	14.56 ± 1.57	55.15 ± 8.16	173.85 ± 33.15	12.37 ± 1.19	43.15 ± 8.8	149 ± 22.06	13.6 ± 1.33	28.11 ± 6.06	146.23 ± 9.75	9.93 ± 0.83	48.37 ± 8.98	167.26 ± 11.51
		**bc**	**b**	**a**	**c**	**bc**	**a**	**bc**	**bc**	**a**	**c**	**bc**	**a**
Free polyamines (pmol mg^–1^ DW)	Agm	2.26 ± 0.28	11.08 ± 2.07	2.99 ± 0.69	2.68 ± 0.67	5.27 ± 1.03	2.9 ± 0.33	2.78 ± 0.41	2.7 ± 0.8	2.59 ± 0.58	2.16 ± 0.68	3.46 ± 0.57	2.25 ± 0.26
		**c**	**a**	**bc**	**bc**	**b**	**bc**	**bc**	**bc**	**bc**	**c**	**bc**	**c**
	Cad	0.16 ± 0.06	0.27 ± 0.09	0.69 ± 0.18	0.12 ± 0.05	0.16 ± 0.05	0.56 ± 0.2	0.11 ± 0.05	0.16 ± 0.07	1.29 ± 0.56	0.06 ± 0.02	0.19 ± 0.07	0.52 ± 0.11
		**c**	**bc**	**a**	**c**	**c**	**ab**	**bc**	**c**	**a**	**c**	**bc**	**a**
	Dap	63.73 ± 7.73	47.15 ± 8.29	27.04 ± 4.19	30.06 ± 2.93	24.68 ± 5.36	33.07 ± 8.25	46.14 ± 5.35	44.19 ± 9.79	55.88 ± 9.99	51.76 ± 7.18	45.52 ± 9.37	5.77 ± 1.29
		**a**	**abcd**	**d**	**cd**	**de**	**bcd**	**abcd**	**abcd**	**ab**	**abc**	**abcd**	**e**
	Hist	0.68 ± 0.14	0.81 ± 0.17	2.29 ± 0.54	1 ± 0.41	1.52 ± 0.51	2.4 ± 0.95	0.53 ± 0.15	0.47 ± 0.1	0.92 ± 0.29	0.71 ± 0.24	1.46 ± 0.51	1.79 ± 0.43
		**c**	**bc**	**ab**	**abc**	**abc**	**a**	**c**	**c**	**abc**	**c**	**abc**	**abc**
	NorSpm	0.6 ± 0.2	0.89 ± 0.12	0.49 ± 0.12	0.35 ± 0.08	0.46 ± 0.16	0.44 ± 0.16	0.66 ± 0.16	1.11 ± 0.29	0.8 ± 0.28	1.4 ± 0.92	1.44 ± 0.67	0.82 ± 0.24
		**ab**	**a**	**ab**	**ab**	**ab**	**b**	**ab**	**ab**	**ab**	**ab**	**ab**	**a**
	Put	52.51 ± 8.31	207.07 ± 21.71	23.53 ± 5.27	49.21 ± 8.91	100.5 ± 6.17	25.64 ± 5.2	12.34 ± 1	36.25 ± 7.87	11.14 ± 2.13	42.78 ± 7.26	102.98 ± 15.93	33.69 ± 5.15
		**c**	**a**	**cde**	**c**	**b**	**cde**	**de**	**cde**	**e**	**cd**	**b**	**cde**
	Spd	12.52 ± 1.9	23.79 ± 2.64	4.7 ± 0.49	14.69 ± 2.62	5.02 ± 0.73	2.87 ± 0.74	2.97 ± 0.5	4.46 ± 1.02	2.19 ± 0.55	4.89 ± 0.57	6.89 ± 0.96	3.08 ± 0.7
		**b**	**a**	**cd**	**b**	**cd**	**cd**	**cd**	**cd**	**d**	**cd**	**c**	**cd**
	Spm	4.27 ± 0.89	8.72 ± 1.08	15.56 ± 1.83	2.55 ± 0.9	4.64 ± 0.73	3.4 ± 0.76	0.86 ± 0.19	2.51 ± 0.61	1.78 ± 0.38	1.04 ± 0.33	4.2 ± 1.11	2.8 ± 0.37
		**c**	**b**	**a**	**cd**	**c**	**cd**	**d**	**cd**	**cd**	**d**	**c**	**cd**

*The data represent mean values ± standard error (S.E.). Different small letters indicate significant differences within variants according to Duncan’s test after ANOVA (P < 0.05).*

The levels of polyamines were also altered differently in both genotypes. LM20 significantly decreased the levels of Dap in those plants grown under D + H7 at eCO_2_ compared to aCO_2_ (Table 1). However, the sensitive-genotype SF29 accumulated Spm under all stress conditions at aCO_2_ but reduced them at eCO_2_ compared with the plants grown at aCO_2_. Altogether, we could conclude that the eCO_2_ activated different stress response strategies among genotypes. Whereas the eCO_2_ downregulated the Lys metabolism and induced the accumulation of phosphate and sulfate in LM20, it changed the carbon and polyamine metabolism to accumulate compatible solutes in SF29 when both were grown under D + H7.

**FIGURE 4:**
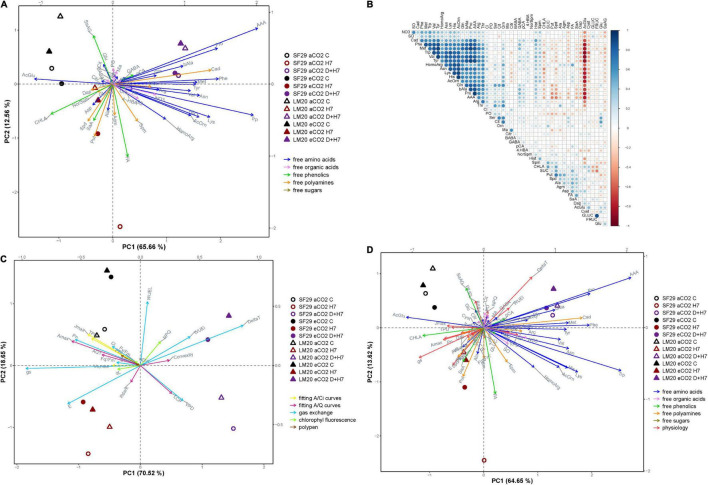
Multivariate statistical analyses of primary and secondary metabolites and plant physiology at the anthesis stage. **(A)** Principal component (PC) analysis of the changes in primary and secondary metabolites; free amino acids, organic acids, phenolics, free polyamines, and carbohydrates represented in blue, pink, green, orange, and brown arrows, respectively, **(B)** correlation matrix among all metabolites, in which the size and intensity of blue (positive) and red (negative) circles correlated with the Pearson’s square-R correlation number, **(C)** PC analysis of the changes in the physiological parameters, and **(D)** PC analysis of the metabolites and physiological parameters together in heat-sensitive (SF29) and heat-tolerant (LM20) genotypes grown under control conditions (C), heat stress at Day 4 (H4), at Day 7 (H7), and combined heat stress and drought at Day 7 (D + H7) at ambient CO_2_ (aCO_2_) or elevated CO_2_ (eCO_2_). The *X*-axis and *Y*-axis represent the PC1 and PC2, respectively, with the percentage of the total variance of the model.

For better visualization of the results, the metabolite data were analyzed using different PCAs and a correlation matrix as shown in Figure 4. The first PCA was done using only the metabolite data (Figure 4A). The *x*-axis represented the PC1 that represented 65.66% of the variance of the model. It mainly separated the plants from both genotypes grown under control conditions and combined stresses (D + H7), independently of the CO_2_ levels. Whereas the control plants were related to the phenolic compound CHLA and the amino acid AcGlu, all the plants grown under D + H7 correlated mainly with the accumulation of many amino acids, including AAA, βAla, and Pro among others, and also the polyamine Cad (Figure 4A). The correlation matrix showed that the changes of the stress-related metabolites, such as βAla, Cad, Met, AcOrn, and Pro, were positively correlated (Figure 4B), so they changed in parallel. It also corroborated the inverse relationship of these compounds with AcGlu. The *y*-axis of Figure 4A represented PC2 with additional 12.56% of the total variance of the model. In this case, it mainly separated LM20 and SF29 grown under control and H7 at aCO_2_, which correlated with the phenolic compounds SaAG and FA, respectively.

To go further, we prepared two additional PCAs: one with the physiological parameters (Figure 4C) and another one with the physiology and metabolites together (Figure 4D). The physiological parameters divided the PC1 (*x*-axis with 70.52% of the total variance of the model) between the plants from the combined stress and the rest, independently of the CO_2_ levels (Figure 4C). However, the most interesting separation was obtained by PC2 (*y*-axis with 18.65% of the total variance of the model) that divided the plants grown under D + H7 between those at aCO_2_ and eCO_2_. Thus, whereas the plants under D + H7 at eCO_2_ positively correlated with WUE_*i*_ and ΔT, and, to less extent, with the convexity of the A/Q curve and NPQ, at aCO_2_, they correlated with LCP and VPD. When the combination between the physiology and the metabolic data was analyzed together (Figure 4D), we could see that the parameters WUE_*i*_ and ΔT were positively correlated with GABA levels and negatively with many gas exchange parameters, such as P_*n*_, E, g_*s*_, and A_*max*_, among others, and the levels of certain polyamines included Spm, Spd, Put, and Dap and the phenolic compound SaA. These results pointed to the GABA and polyamines as very important metabolites regulating stress response and tolerance of spring wheat under combined stresses and different CO_2_ levels.

### Elevated CO_2_ Only Improved the Grain Yield-Related Parameters in Spring Wheat Under Control but Not Under Stress

**FIGURE 5:**
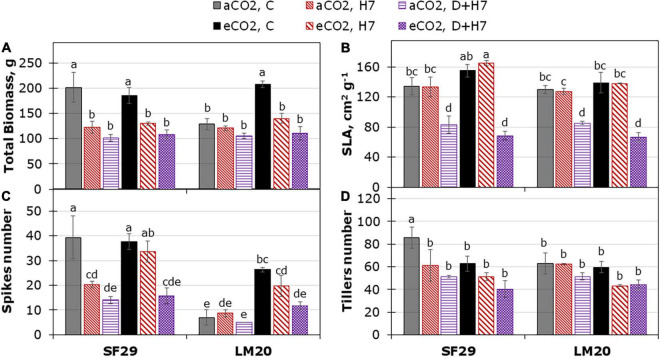
Yield-related traits from destructive harvest during the anthesis stage. **(A)** Total biomass dry weight, **(B)** specific leaf area (SLA), and the number of **(C)** spikes and **(D)** tillers per plant in heat-sensitive (SF29) and heat-tolerant (LM20) genotypes grown under control conditions (C), heat stress at Day 4 (H4), at Day 7 (H7), and combined heat stress and drought at Day 7 (D + H7) at ambient CO_2_ (aCO_2_) or elevated CO_2_ (eCO_2_). The data represent mean values ± standard error (S.E.) (*n* = 3). Different small letters indicate significant differences within variants according to Duncan’s test after ANOVA (*p* < 0.05).

**FIGURE 6:**
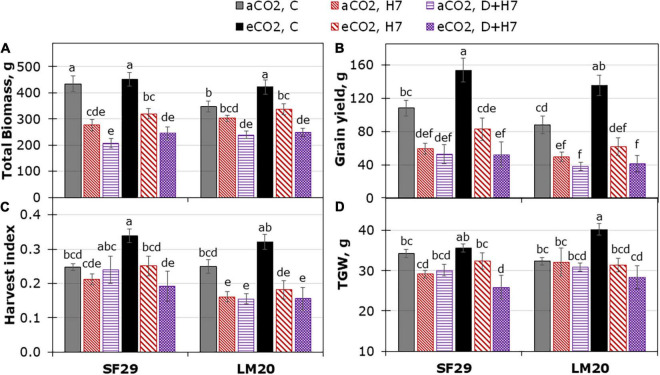
Yield-related traits during the ripening stage. **(A)** Total biomass (DW), **(B)** grain yield **(C)** harvest index, and **(D)** thousand-grain weight (TGW) per plant in heat-sensitive (SF29) and heat-tolerant (LM20) genotypes grown under control conditions (C), heat stress at Day 7 (H7) and combined heat stress and drought at Day 7 (D + H7) at ambient CO_2_ (aCO_2_) or elevated CO_2_ (eCO_2_). The data represent mean values ± S.E. (*n* = 8). Different small letters indicate significant differences within variants according to Duncan’s test after ANOVA (*p* < 0.05).

Finally, we decided to evaluate the impact of the different stress conditions and the interaction with aCO_2_ or eCO_2_ in the final yield of the plants. For that, a destructive harvest was performed during the anthesis (Figure 5), and the ripening stage (Figure 6 and Supplementary Figure 5). The plant yield did not change due to the triple interaction between genotype, treatment, and CO_2_, and the main effect was induced by the treatment (*p*-value lower than 0.001) (Supplementary Table 2). However, the CO_2_ factor interacted with genotype or treatment for some variables such as spike number at anthesis (aSPKN), or biomass-related parameters such as SLA, and the DW of leaf and total biomass at the ripening stage, respectively. In general, SF29 was a more productive genotype than LM20 under control conditions at aCO_2_ (Figure 5), with a higher number and weight of tillers and spikes at the anthesis and ripening stages, respectively (Figure 5 and Supplementary Figure 5). Contrarily, LM20 had heavier leaves than SF29 (Supplementary Figure 5). At anthesis, the eCO_2_ improved the total biomass and the spike number of the heat-tolerant genotype (LM20) under control conditions (Figures 5A,C). However, in the heat-sensitive genotype (SF29), the application of eCO_2_ improved the SLA and the number of spikes under heat stress compared to the plants grown at aCO_2_ (Figures 5B,C).

**FIGURE 7:**
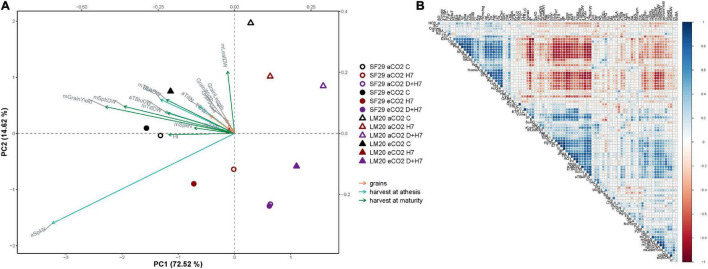
Multivariate statistical analyses of yield-related parameters. **(A)** Principal component (PC) analysis of the changes in yield-related parameters represented grain yield, the harvest at the anthesis and the ripening stage in orange, blue, and green arrows, respectively, and **(B)** correlation matrix between the metabolites and physiological and yield-related parameters, in which the size and intensity of blue (positive) and red (negative) circles correlated with the Pearson’s square-R correlation number in heat-sensitive (SF29) and heat-tolerant (LM20) genotypes grown under control conditions (C), heat stress at Day 7 (H7) and combined heat stress and drought at Day 7 (D + H7) at ambient CO_2_ (aCO_2_) or elevated CO_2_ (eCO_2_). The *X*-axis and *Y*-axis represent the PC1 and PC2, respectively, with the percentage of the total variance of the model.

When the production was evaluated during the ripening stage, no eCO_2_-mitigation effect was observed in any plant grown under stress (Figure 6 and Supplementary Figure 5). The eCO_2_ only improved the yield-related parameters when the plants were grown under control conditions. Total biomass was significantly higher in SF29 than LM20 under control conditions at aCO_2_, and the application of eCO_2_ increased this parameter in LM20 but not in SF29. Higher total biomass at eCO_2_ was more due to the improvement of the weight in the reproductive organs rather than in the leaves, as confirmed by a significantly higher grain area, width and length, and TGW observed in LM20 compared to SF29 (Figures 6B,D and Supplementary Figure 5). Contrarily, SF29 improved the harvest index, mainly because of the higher weight of the spikes (Figure 6C and Supplementary Figure 5C). The obtained results were supported by additional PCA, in which the production parameters were analyzed (Figure 7A). The PC1 (*x*-axis) was able to represent 72.52% of the total variance of the model. There, we observed that almost all yield-related parameters were related to SF29 at control conditions at both aCO_2_ and eCO_2_ and to LM20 only at eCO_2_ (Figure 7A). However, the PC2 (*y*-axis, 14.62%) showed that LM20 plants had higher leaf weight under control conditions at aCO_2_, whereas, under stress, SF29 plants reduced (were negatively correlated) this parameter. Altogether, it was clear that the improvement in grain yield induced by eCO_2_ is genotype dependent when it was grown under optimal conditions, helping more the less-productive genotype (in this case, LM20). However, the application of eCO_2_ could have no effect or rather a negative effect on the sensitive genotypes when they suffer combined stresses such as heat and drought.

Finally, a matrix correlation including all data was performed (Figure 7B). There, we observed that WUE_*i*_ presented an inverse correlation with almost all photosynthetic and grain yield-related parameters, which were also negatively correlated with several amino acids, such as AAA, Pro, βAla, Lys, and AcOrn, among others, and the polyamine Cad. Only the amino acid AcGlu presented a positive correlation with photosynthetic and yield-related parameters (Figure 7B). Altogether, it is clear that the synthesis of metabolites as a strategy to deal with the stress by enhancing WUE could condition other physiological processes, including the fluorescence-related parameter and the final yield in spring wheat.

## Discussion

Drought and heat waves commonly co-occur in many wheat-growing regions, causing significant crop losses. In the plants, both stresses independently or in combination affect plant physiology and grain yield (Qaseem et al., 2019). However, the level of exacerbated damage on physiological, metabolic, and yield traits under a combination of heat and drought stress depends on the plant developmental stage (Hlaváčová et al., 2018). Moreover, the impact of eCO_2_ on mitigating the negative effect of drought and heat stress varies between cultivars due to their different levels of susceptibility to the induced stress (Schmidhuber and Tubiello, 2007). In this work, we investigated the impact of the combined heat and drought stress in spring wheat and the possible mitigation effect induced by eCO_2_. We selected two spring wheat genotypes: one heat sensitive (SF29) and another heat tolerant (LM20). The study was performed during anthesis because it is known that heat stress with temperatures over 31°C during this developmental stage reduced grain yield in wheat (Porter and Gawith, 1999). Different responses at morphological, physiological, and metabolic levels were observed between both genotypes regarding the stress conditions and CO_2_ treatment.

Little is known about the combined effects of heat and drought stress on crops, with most studies reporting very severe effects on crop growth and productivity (Reviewed by Sehgal et al., 2018). Recent studies have shown that, whereas heat stress affects more the physiology of the plant, drought reduces the production of wheat (Qaseem et al., 2019). The same work also showed that the combination of heat and drought stress reduced the membrane structure, chlorophyll and protein molecules, and plant yield (Qaseem et al., 2019). Here, our results showed that the combination of heat and drought stress highly affected the photosynthetic and fluorescence-related parameters in both genotypes (Figures 1–3 and Supplementary Figures 1, 2). However, as shown by the statistical analysis (Supplementary Table 2), the bigger impact was in the dark phase of the plant photosynthesis. Interestingly, when eCO_2_ was applied, the heat-sensitive genotype SF29 increased P_*n*_, C_*i*_, and other photosynthetic parameters, such as J_*max*_, TPU, and A_*max*_ under combined stress, but not LM20 (Figures 1, 2). It is known that photosynthesis-related parameters can reflect the thermotolerance of the plants (Wahid et al., 2007). However, no significant differences in the ΔT were observed in D + H7 stressed plants when they were grown at aCO_2_ or eCO_2_ (Figure 1F). Contrary, whereas LM20 reduced the LRWC under D + H7 at eCO_2_, SF29 maintained it (Supplementary Figure 3B). It could be that a higher synthesis of the photoassimilates GLUC and FRUC observed in SF29 but not in LM20 improved the stress tolerance of the plants in two ways: (i) as compatible solutes to maintain the water balance, or (ii) as carbon skeletons to synthesize free amino acids (De Diego et al., 2013).

The heat-sensitive SF29 had significantly higher levels of GABA and βAla, and lower content of Cit, Cyst, Orn, and the polyamine Spm when the plants were grown under D + H7 at eCO_2_ compared to aCO_2_ (Table 1). However, they did not change the content of Glu. Contrarily, LM20 significantly reduced Glu, Hist, HomoArg, and Lys, and the polyamine Dap, and it also tended to have lower levels of Cad. It is well-known that GABA can be synthesized *via* Glu or polyamines (Podlešáková et al., 2019). Here, there were no changes in Glu, and the reduced levels of Spm and its precursors Cit and Orn pointed to the upregulation of polyamine catabolism as the main pathway contributing to the GABA accumulation in SF29 under D + H7 at eCO_2_. Thus, the plants could activate their antioxidative response induced by the H_2_O_2_ accumulation produced in this pathway (Podlešáková et al., 2019). It could also explain the accumulation of βAla, which is another final product of the polyamine pathway. Many plants accumulated the non-proteic amino acid βAla under stress conditions, including heat stress and drought (reviewed by Parthasarathy et al., 2019). Besides, it used to be converted to βAla betaine, which is a compatible solute that enhances the osmotic potential of the plants and, hence, the tolerance against stress. Finally, it is worth mentioning that GABA acts as a signal molecule regulating physiological processes including stomata closure and WUE (Xu et al., 2021). Thus, GABA could also improve the photosynthesis efficiency in SF29 under D + H7 at eCO_2_. Altogether, we could say that the application of eCO_2_ improved the SF29 resilience by a better osmotic response and a photosynthetic capacity. Similar results were described by Ulfat et al. (2021), in which the application of eCO_2_ reduced the negative impact of drought in wheat mainly due to the maintenance of higher total antioxidants potential, which enables the plants to scavenge the ROS production and maintain an optimum photosynthetic rate.

In LM20 plants, eCO_2_ reduced the accumulation of many metabolites involved in the Lys metabolism, including Lys, HomoArg, and the polyamine Cad (Table 1). Cad can be synthesized *via* Lys and HomoArg (Jancewicz et al., 2016). Under stress conditions, plants accumulate and transport Cad through the different organs of the plants (Jancewicz et al., 2016). However, it is still unclear if its accumulation induced sensitivity or tolerance. One theory is that Cad acts as an antioxidative response of the plants to deal with stress, as mentioned by Marchetti et al. (2019). Besides, it has been reported that eCO_2_ application induced many hormonal changes in the plants (Vicente et al., 2019). In this regard, it was shown that eCO_2_ reduced the accumulation of ethylene in wheat plants (Vicente et al., 2019). Besides, ethylene regulates the production of Cad under heat stress conditions (Shevyakova et al., 2001) and reduces the photosynthesis and photochemical efficiency under severe water deficit (Yang et al., 2014). Thus, eCO_2_ could reduce the ethylene accumulation in LM20 as a defense mechanism against the combined stresses to reduce the Cad accumulation and to maintain fluorescence-related parameters (Figure 3). Together with that, the application of eCO_2_ also significantly increased the levels of phosphate and sulfate in LM20 under combined stress (D + H7) (Table 1). The application of phosphate has been reported to mitigate the negative effect of heat stress by enhancing plant photosynthesis, WUE, and also yield (reviewed by Bechtaoui et al., 2021). Besides, the sulfates also can improve overall plant performance (Usmani et al., 2020). Thus, eCO_2_ activated different mechanisms to increase the WUE of the plants in both the heat-tolerant and sensitive genotypes to deal with the negative effect of the combined stresses.

As mentioned above, eCO_2_ induced changes in WUE_*i*_ and WUE_*leaf*_ in both genotypes, especially under control conditions and combined stresses (Figure 1E and Supplementary Figure 3A). This effect was higher in LM20 than in SF29. WUE is expressed as the capacity of a crop to produce biomass per unit of water evapotranspiration and, hence, the major component of yield (Rizza et al., 2012). According to this, we expected an increase of grain yield-related parameters at eCO_2_. Our results showed that eCO_2_ did not modify the yield but enhanced the total biomass, TGW, and grain dimension in the less-productive genotype LM20 but not in SF29 under control conditions (Figures 5, 6 and Supplementary Figure 5). It can be because, under stress conditions, the combination of eCO_2_ with other limiting factors such as heat and drought could harm yield as described by Amthor (2001). Thus, heat stress reduced the grain yield as a consequence of the reduced accumulation of photoassimilates for grain development and shortened the grain-filling duration, a negative effect that cannot be mitigated by eCO_2_ (Zhang et al., 2018). However, our results showed that the photosynthesis-related parameters and the accumulation of GLUC and FRUC in SF29 were improved under combined stress at eCO_2_, but these changes rather reduced the yield under these growth conditions (Figures 5–7 and Supplementary Figure 5). Another explanation can be the effect of eCO_2_ diminished on grain yield under heat stress at anthesis due to grain abortion (Chavan et al., 2019). In this regard, we showed that eCO_2_ did not reduce the ΔT of the plants, pointing to the high temperature as one of the main limitations to improve the plant yield. Additionally, a recent study has reported that the higher assimilation rate under eCO_2_ in wheat genotypes with lower osmotic potential was used to maintain the osmotic adjustment rather than the yield-related traits in response to drought stress at anthesis (Shokat et al., 2021). However, a previous study performed in 64 wheat cultivars did not find the correlation between the photosynthetic capacity of the flag leaf and grain yield or plant biomass at pre-anthesis as a consequence of natural variation between existing wheat cultivars (Driever et al., 2014). In our work, an improvement of the assimilation rate and a higher accumulation of compatible solutes such as carbohydrates and amino acids observed in SF29 pointed to this genotype as a resilient genotype that increases the photosynthesis to improve the synthesis of these metabolites involved in the maintenance of a higher water balance (higher LRWC than LM20, Supplementary Figure 3B). Thus, we could conclude that eCO_2_ cannot alleviate the losses in grain yield induced by combined heat and drought stress during anthesis in spring wheat, despite a better water balance.

To ensure the yield, plants need a balance between the source (leaf photosynthetic potential and the levels of assimilates) and the available sink capacity (grain yield). In our study, SUC was only quantified in SF29 under all growth conditions at aCO_2_, especially under control conditions. SUC is the end product of photosynthesis and the primary sugar transported in the phloem of most plants (Ruan, 2014). Under well-watered conditions, wheat plants with higher levels of SUC in the flag leaf are expected to have better production (Ahmed et al., 2020). This could explain the better yield-related parameters in SF29 (Figures 5–7 and Supplementary Figure 5). However, at eCO_2_, the differences between both genotypes disappeared, ending both with better grain yield under control conditions (Figure 6B). This gain was not due to the SUC, so both genotypes reduced the content under the detection level (Table 1). One possibility is the changes of GLUC and FRUC that happened at eCO_2_ influenced the yield parameters (Table 1). However, an opposite response was observed among both genotypes. Whereas SF29 significantly increased GLUC and FRUC levels, LM20 reduced them. Additionally, SF29 only increased the weight of spikes, whereas LM20 had heavier spikes and produced bigger grains at eCO_2_. El Habti et al. (2020) showed that both fructose and glucose could determine the final grain weight in wheat under combined heat stress and drought. However, the influence of these two soluble carbohydrates was genotype dependent. Glucose and fructose are the first substrates in the starch biosynthesis pathway, and the starch accumulation determines the grain filling. Besides, it has been proposed that sink strength (grain capacity) can be the limiting factor in starch accumulation and grain filling in favorable environments (El Habti et al., 2020). In addition, the varied responses among winter wheat cultivars were depending on their tolerance in response to heat stress at anthesis and mid-grain filling stages to maintain photosynthetic activity and grain yield (Mirosavljević et al., 2021). Altogether, we could conclude that, under control conditions, the eCO_2_ differently affected both genotypes. Whereas it improved the grain-filling capacity of LM20, it could reduce the number of abortions in the spike (higher weight) in both genotypes, and this effect was not related to the GLUC and FRUC contents.

## Conclusion

In conclusion, the selected wheat genotypes showed different physiological responses to stress in combination with and without eCO_2_ during anthesis. The heat-sensitive genotype (SF29) improved the dark phase of the photosynthesis and accumulated metabolites, such as GLUC, FRUC, βAla, and GABA, which are considered compatible solutes. It helped to improve the antioxidant response of the plant, maintain the water balance, and increase the WUE as a tolerance strategy. However, heat-tolerant genotype (LM20) downregulated the Lys metabolism, most probably as a reduced ethylene synthesis to maintain or improve the fluorescence-related parameters and the WUE. This genotype also accumulated higher content of phosphate and sulfate to mitigate the stress. However, these strategies did not help the genotypes to improve the yield losses of the plants grown under stress conditions, especially under combined stresses at eCO_2_. However, eCO_2_ improves photosynthesis, WUE, and spring wheat yield under non-stress conditions, enhancing spike weight in both genotypes and the size of the grains in the less-productive one. Our results suggested that the photosynthetic/source activities are not entirely related to the spring wheat yield, and that there might be other sink limitations that required further investigation to develop robust genotypes for the future.

## Data Availability Statement

The original contributions presented in the study are included in the article/Supplementary Material, further inquiries can be directed to the corresponding authors.

## Author Contributions

LA designed and performed the experiment. TM and CP assisted with conducting the experiment and harvesting. OV, SĆ, and PT performed the targeted metabolomics analysis, metabolite quantification, and wrote the metabolomic part. NŠ performed the statistical analysis. LA and ND analyzed the data and wrote the manuscript draft. BW, ER, and C-OO contributed to the original concept of the project and supervised the study. All authors contributed to the manuscript and approved the submitted version.

## Conflict of Interest

The authors declare that the research was conducted in the absence of any commercial or financial relationships that could be construed as a potential conflict of interest. The reviewer PC declared a shared affiliation with one of the authors ER to the handling editor at the time of the review.

## Publisher’s Note

All claims expressed in this article are solely those of the authors and do not necessarily represent those of their affiliated organizations, or those of the publisher, the editors and the reviewers. Any product that may be evaluated in this article, or claim that may be made by its manufacturer, is not guaranteed or endorsed by the publisher.

## Supplementary Material

Supplementary Figure 1 Light response curves. **(A)** Net photosynthetic rate (P_*n*_), **(B)** intercellular CO_2_ (C_*i*_), **(C)** stomatal conductance (g_*s*_), **(D)** transpiration rate (E) and **(E)** difference between leaf and air cuvette temperature (ΔT) in heat-sensitive (SF29) and heat-tolerant (LM20) genotypes grown under control conditions (C), heat stress at Day 4 (H4), at Day 7 (H7), and combined heat stress and drought at Day 7 (D + H7) at ambient CO_2_ (aCO_2_) indicated as dashed lines or elevated CO_2_ (eCO_2_) indicated as solid lines. The data represent mean values ± standard error (S.E.) (*n* = 6–8).

Supplementary Figure 2 Chlorophyll fluorescence parameters from light response curves. **(A)** Operating efficiency of PSII (F_*q*_′/F_*m*_′) and **(B)** electron transport rate (ETR) in heat-sensitive (SF29) and heat-tolerant (LM20) genotypes grown under control conditions (C), heat stress at Day 4 (H4), at Day 7 (H7), and combined heat stress and drought at Day 7 (D + H7) at ambient CO_2_ (aCO_2_) indicated as dashed lines or elevated CO_2_ (eCO_2_) indicated as solid lines. The data represent mean values ± standard error (S.E.) (*n* = 6–8).

Supplementary Figure 3 The water status in plants. **(A)** Instantaneous water use efficiency (WUE_*leaf*_), and **(B)** leaf relative water content (LRWC) in heat-sensitive (SF29) and heat-tolerant (LM20) genotypes grown under control conditions (C), heat stress at Day 4 (H4), at Day 7 (H7), and combined heat stress and drought at day 7 (D + H7) at ambient CO_2_ (aCO_2_) or elevated CO_2_ (eCO_2_). The data represent mean values ± standard error (S.E.) (*n* = 6–8). Different small letters indicate significant differences within variants according to Duncan’s test after ANOVA (*p* < 0.05).

Supplementary Figure 4 Fitted parameters from the light response curves; **(A)** light compensation point (LCP) and **(B)** convexity of the curve (θ), and leaf reflectance represented as **(C)** photochemical reflectance index (PRI) in heat-sensitive (SF29) and heat-tolerant (LM20) genotypes grown under control conditions (C), heat stress at Day 4 (H4), at Day 7 (H7) and combined heat stress and drought at Day 7 (D + H7) at ambient CO_2_ (aCO_2_) or elevated CO_2_ (eCO_2_). The data represent mean values ± standard error (S.E.) (*n* = 6–8). Different small letters indicate significant differences within variants according to Duncan’s test after ANOVA (*p* < 0.05).

Supplementary Figure 5 Morphological and grain traits from destructive harvest at ripening. **(A,B)** Number of spikes and tillers per plant, **(C–E)** dry weight of spikes, tillers, and leaves per plant and **(F–H)** Grain dimensions including area, width, and length in heat-sensitive (SF29) and heat-tolerant (LM20) genotypes grown under control conditions (C), heat stress at Day 4 (H4), at Day 7 (H7), and combined heat stress and drought at Day 7 (D + H7) at ambient CO_2_ (aCO_2_) or elevated CO_2_ (eCO_2_). The data represent mean values ± standard error (S.E.) (*n* = 6–8). Different small letters indicate significant differences within variants according to Duncan’s test after ANOVA (*p* < 0.05).

## Abbreviations

Δ T or DeltaT: difference between leaf and air cuvette temperature
4-HBA: 4-hydroxybenzoic acid
AAA: 2-aminoadipic acid
AcGlu: N-acetyl glutamate
AcOrn: N-acetylornithine
Agm: agmantine
Ala: alanine
aLeafDW: leaf dry weight at anthesis
A_*max*_: maximum net CO_2_ assimilation rate at light saturation
Arg: arginine
Asn: Asparagine
Asp: aspartic acid
aSpkDW: spike dry weight at anthesis
aSpkN: spikes number at anthesis
aTBioDW: total biomass dry weight at anthesis
aTBioFW: total biomass fresh weight at anthesis
aTillDW: tiller dry weight at anthesis
aTillN: tillers number at anthesis
BABA: β -aminobutyric acid
β Ala: β -alanine
Cad: cadaverine
CHLA: chlorogenic acid
C_*i*_: intercellular CO_2_
Cit: citrulline
Citr: citrate
Cl: chloride
Cyst: cysteine
Dap: 1,3-diaminopropane
E: transpiration rate
ETR: electron transport rate
FA: ferulic acid
F_*q*_ ′ /F_*m*_ ′ or Y-LC: operating efficiency of PSII on light-adapted leaves from light response curve
FRUC: fructose
F_*v*_/F_*m*_: maximum quantum efficiency of PSII on dark-adapted leaves
GABA: γ -aminobutyric acid
Gln: glutamine
GLUC: glucose
Glu: glutamic acid
g_*s*_: stomatal conductance
HI: harvest index
His: histidine
Hist: histamine
HomoArg: homoarginine
J_*max*_: photosynthetic electron transport rate
LA: leaf area
LCP: light compensation point
LRWC: leaf relative water content
Lys: lysine
Ma: malate
Met: methionine
mGrainYield: grain yield at maturity
mLeafDW: leaf dry weight at maturity
mSpkDW: spikes dry weight at maturity
mSpkN: spikes number at maturity
mTBioDW: total biomass dry weight at maturity
mTillDW: tillers dry weight at maturity
mTillN: tillers number at maturity
NDVI: normalized difference vegetation index
NO_3_: nitrate
NorSpd: norspermidine
NPQ: non-photochemical quenching
Orn: ornithine
pCA: *p*-coumaric acid
Phe: phenylalanine
P_*n*_: net photosynthetic rate
PO: phosphate
PRI: photochemical reflectance index
Pro: proline
Put: putrescine
q_*L*_: fraction of open PSII centers
R_*d*_-LC: dark respiration rate from light curve
SaA: salicylic acid
SaAG: salicylic acid glucoside
Ser: serine
SLA: specific leaf area
SO: sulfate
Spd: spermidine
Spm: spermine
SUC: sucrose
TGW: thousand grain weight
Thr: threonine
TPU: triose-phosphate utilization
Trp: tryptophan
Tyr: tyrosine
Val: valine
V_*c*,max_: carboxylation rate by Rubisco
WUE_*i*_: intrinsic water use efficiency
WUE_*Leaf*_: instantaneous leaf water use efficiency
α or AQY: apparent quantum yield of CO_2_ assimilation
θ or convex: convexity of the curve.
