# Supplementary figures and images for: Elevated CO2 Improves the Physiology but Not the Final Yield in Spring Wheat Genotypes Subjected to Heat and Drought Stress During Anthesis
Source: Front Plant Sci. 2022 Mar 7;13:824476. doi: 10.3389/fpls.2022.824476 (PMC8940247; doi:10.3389/fpls.2022.824476)

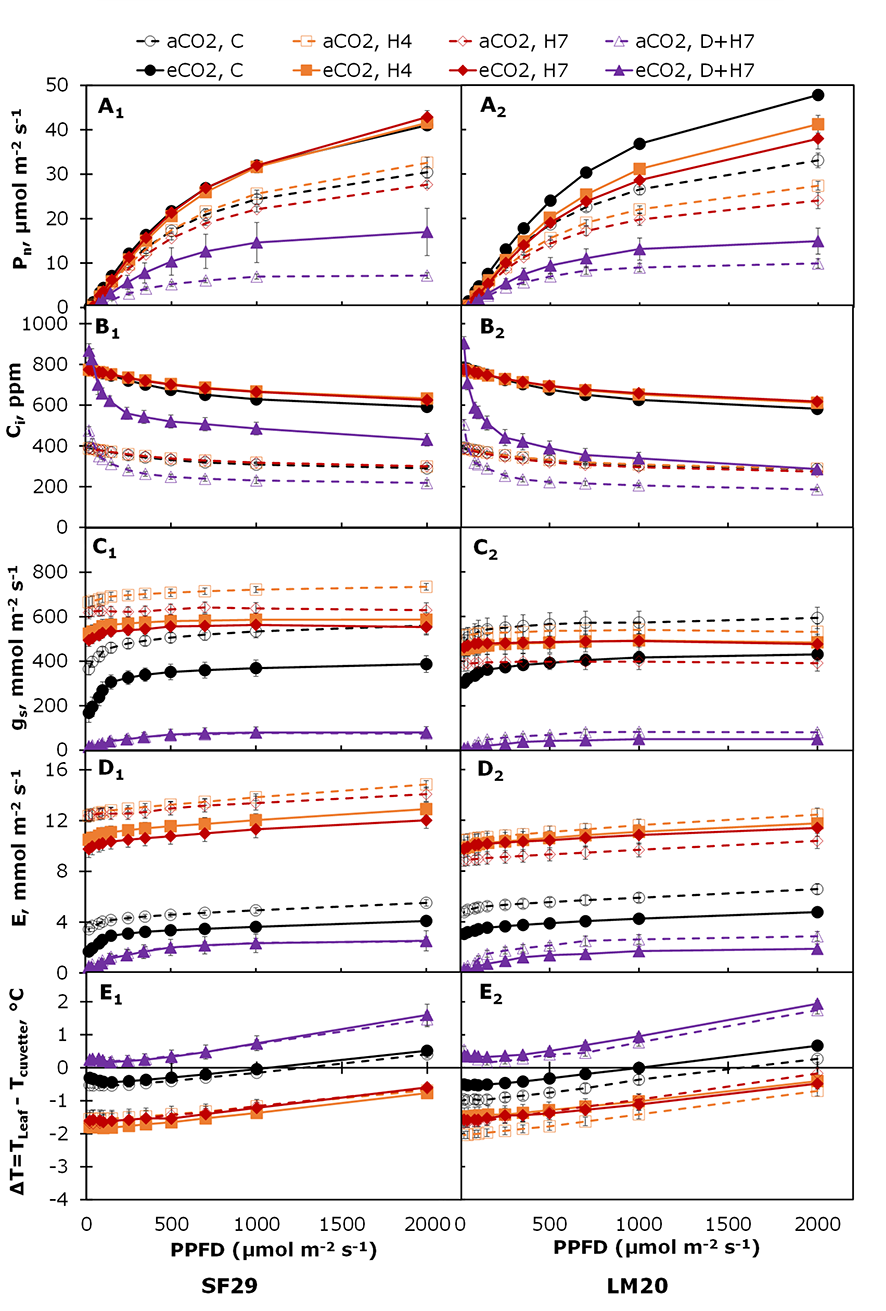

Supplement: Supplementary Figure 1 — Light response curves. (A) Net photosynthetic rate (Pn), (B) intercellular CO2 (Ci), (C) stomatal conductance (gs), (D) transpiration rate (E) and (E) difference between leaf and air cuvette temperature (ΔT) in heat-sensitive (SF29) and heat-tolerant (LM20) genotypes grown under control conditions (C), heat stress at Day 4 (H4), at Day 7 (H7), and combined heat stress and drought at Day 7 (D + H7) at ambient CO2 (aCO2) indicated as dashed lines or elevated CO2 (eCO2) indicated as solid lines. The data represent mean values ± standard error (S.E.) (n = 6–8). [file Image_1.TIF]

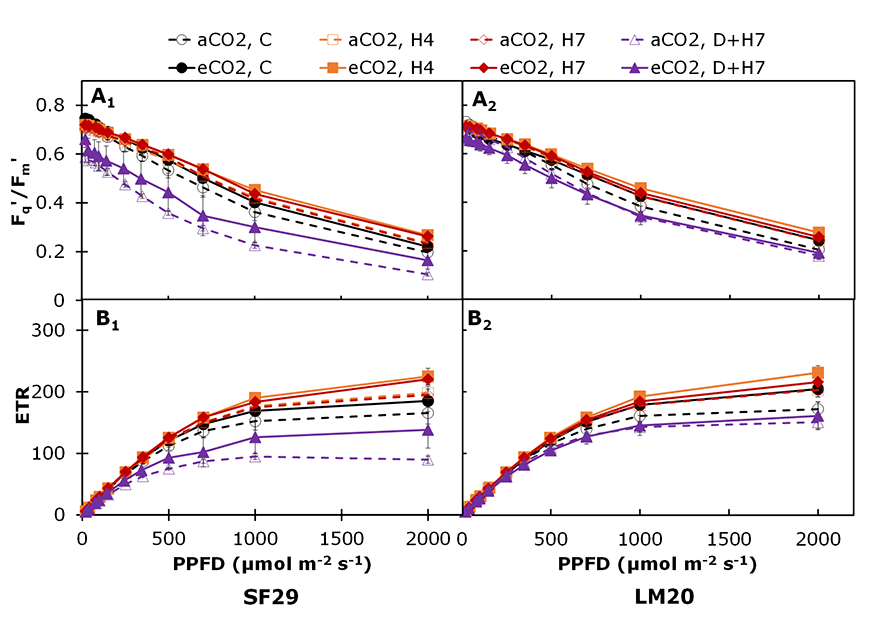

Supplement: Supplementary Figure 2 — Chlorophyll fluorescence parameters from light response curves. (A) Operating efficiency of PSII (Fq′/Fm′) and (B) electron transport rate (ETR) in heat-sensitive (SF29) and heat-tolerant (LM20) genotypes grown under control conditions (C), heat stress at Day 4 (H4), at Day 7 (H7), and combined heat stress and drought at Day 7 (D + H7) at ambient CO2 (aCO2) indicated as dashed lines or elevated CO2 (eCO2) indicated as solid lines. The data represent mean values ± standard error (S.E.) (n = 6–8). [file Image_2.TIF]

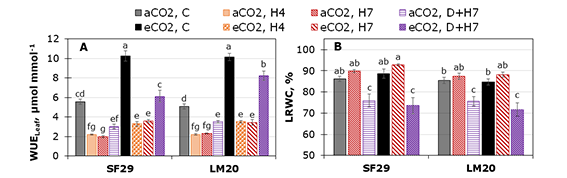

Supplement: Supplementary Figure 3 — The water status in plants. (A) Instantaneous water use efficiency (WUEleaf), and (B) leaf relative water content (LRWC) in heat-sensitive (SF29) and heat-tolerant (LM20) genotypes grown under control conditions (C), heat stress at Day 4 (H4), at Day 7 (H7), and combined heat stress and drought at day 7 (D + H7) at ambient CO2 (aCO2) or elevated CO2 (eCO2). The data represent mean values ± standard error (S.E.) (n = 6–8). Different small letters indicate significant differences within variants according to Duncan’s test after ANOVA (p < 0.05). [file Image_3.tif]

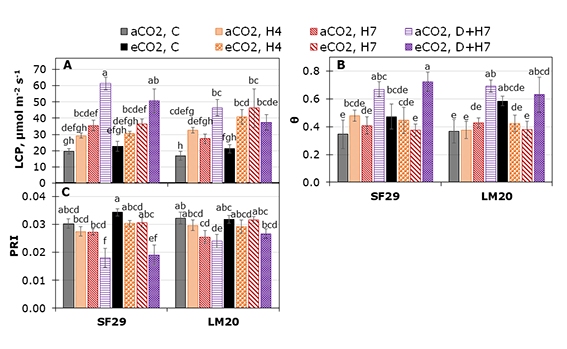

Supplement: Supplementary Figure 4 — Fitted parameters from the light response curves; (A) light compensation point (LCP) and (B) convexity of the curve (θ), and leaf reflectance represented as (C) photochemical reflectance index (PRI) in heat-sensitive (SF29) and heat-tolerant (LM20) genotypes grown under control conditions (C), heat stress at Day 4 (H4), at Day 7 (H7) and combined heat stress and drought at Day 7 (D + H7) at ambient CO2 (aCO2) or elevated CO2 (eCO2). The data represent mean values ± standard error (S.E.) (n = 6–8). Different small letters indicate significant differences within variants according to Duncan’s test after ANOVA (p < 0.05). [file Image_4.TIF]

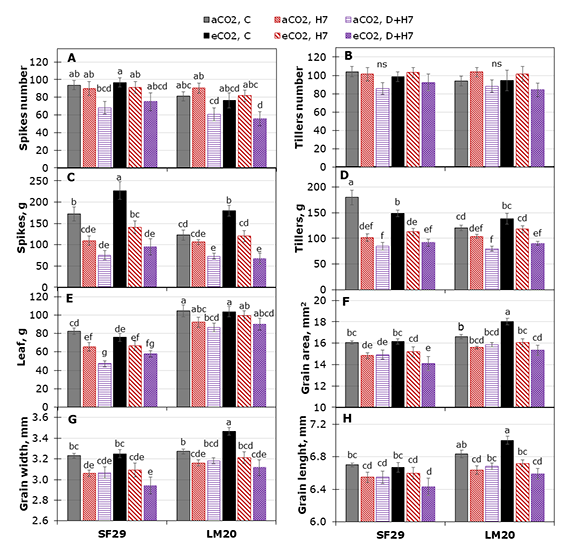

Supplement: Supplementary Figure 5 — Morphological and grain traits from destructive harvest at ripening. (A,B) Number of spikes and tillers per plant, (C–E) dry weight of spikes, tillers, and leaves per plant and (F–H) Grain dimensions including area, width, and length in heat-sensitive (SF29) and heat-tolerant (LM20) genotypes grown under control conditions (C), heat stress at Day 4 (H4), at Day 7 (H7), and combined heat stress and drought at Day 7 (D + H7) at ambient CO2 (aCO2) or elevated CO2 (eCO2). The data represent mean values ± standard error (S.E.) (n = 6–8). Different small letters indicate significant differences within variants according to Duncan’s test after ANOVA (p < 0.05). [file Image_5.TIF]
