# Supplementary material for: Elevated CO2 Improves the Physiology but Not the Final Yield in Spring Wheat Genotypes Subjected to Heat and Drought Stress During Anthesis
Source: Front Plant Sci. 2022 Mar 7;13:824476. doi: 10.3389/fpls.2022.824476 (PMC8940247; doi:10.3389/fpls.2022.824476)
Supplement: Supplementary file 6 [file Table_1.pdf]

Supplementary Table S1. Maximum quantum efficiency of PSII photochemistry (F_v/F_m) values at heat stress (3rd day at 40°C) and recovery phase (7th day after removing heat stress) in 24 Nordic wheat genotypes. The data represent mean values ± S.E. (n = 8).

**Genotype**	**F_v/F_m at heat stress**	**F_v/F_m at recovery**
SF38	0.519 ± 0.052	0.740 ± 0.023
**SF29**	0.562 ± 0.038	0.731 ± 0.029
SF20	0.616 ± 0.038	0.783 ± 0.004
AL5	0.630 ± 0.035	0.790 ± 0.003
NS27	0.646 ± 0.025	0.771 ± 0.016
SF9	0.653 ± 0.022	0.779 ± 0.007
NS16	0.656 ± 0.036	0.792 ± 0.003
SF1	0.657 ± 0.025	0.748 ± 0.021
LM17	0.665 ± 0.028	0.761 ± 0.011
LM26	0.675 ± 0.034	0.761 ± 0.013
LM11	0.675 ± 0.034	0.762 ± 0.006
LM62	0.677 ± 0.028	0.798 ± 0.004
NS19	0.680 ± 0.029	0.795 ± 0.002
LM19	0.682 ± 0.028	0.748 ± 0.012
KU10	0.688 ± 0.018	0.744 ± 0.013
SF8	0.689 ± 0.013	0.786 ± 0.003
SF11	0.698 ± 0.020	0.716 ± 0.030
**LM20**	0.707 ± 0.013	0.780 ± 0.006
NS25	0.709 ± 0.020	0.803 ± 0.001
GN5	0.714 ± 0.011	0.776 ± 0.010
NS35	0.716 ± 0.005	0.772 ± 0.013
NS3	0.718 ± 0.017	0.799 ± 0.002
NS33	0.744 ± 0.004	0.784 ± 0.013
NS36	0.753 ± 0.005	0.801 ± 0.001
