# Supplementary material for: Elevated CO2 Improves the Physiology but Not the Final Yield in Spring Wheat Genotypes Subjected to Heat and Drought Stress During Anthesis
Source: Front Plant Sci. 2022 Mar 7;13:824476. doi: 10.3389/fpls.2022.824476 (PMC8940247; doi:10.3389/fpls.2022.824476)
Supplement: Supplementary file 7 [file Table_2.pdf]

Supplementary Table S2. Three-way ANOVA of physiological parameters and yield-related traits between genotype (G- SF29 or LM20), CO_2 levels (aCO_2 or eCO_2) and treatments (T- heat stress for 4 days (H4), heat stress for 7 days (H7) or combined drought and heat stress (D+H7)).

**Traits**	**G**	**CO_2**	**T**	**G × CO_2**	**G × T**	**CO_2× T**	**G × CO_2 × T**
P_n	0.162	**<0.001**	**<0.001**	0.153	0.058	**0.003**	**<0.001**
C_i	**<0.001**	**<0.001**	**<0.001**	0.348	**<0.001**	**0.002**	**0.009**
g_s	**<0.001**	**0.003**	**<0.001**	0.669	**0.011**	0.076	**<0.001**
E	**0.042**	**0.001**	**<0.001**	0.319	**0.004**	0.123	**<0.001**
ΔT	**0.016**	0.236	**<0.001**	0.676	0.524	0.344	0.492
WUE_i	**<0.001**	**<0.001**	**<0.001**	0.525	0.074	**<0.001**	0.100
WUE_L	0.064	**<0.001**	**<0.001**	0.851	**0.010**	**<0.001**	0.135
VPD	**<0.001**	0.242	**<0.001**	0.075	0.124	**0.019**	0.484
$V_{c,max}$	**<0.001**	**<0.001**	**<0.001**	0.889	**0.005**	**<0.001**	0.223
J_{max}	0.084	**0.001**	**<0.001**	**0.046**	0.881	**<0.001**	**0.014**
TPU	0.378	**<0.001**	**<0.001**	0.138	0.284	**<0.001**	0.131
A_{max}	0.162	**<0.001**	**<0.001**	0.379	0.060	**0.024**	**<0.001**
AQY	0.815	**<0.001**	**<0.001**	0.067	0.680	0.281	0.107
R_{dark}-LC	0.208	**0.002**	**<0.001**	0.782	0.120	0.142	0.405
LCP	0.202	0.178	**<0.001**	0.223	0.055	0.069	0.833
Convexity	0.965	0.398	**<0.001**	0.860	0.474	0.089	0.667
PRI	0.061	0.108	**<0.001**	0.937	**0.007**	0.836	0.722
F_q'/F_m'	**0.012**	**0.007**	**<0.001**	0.626	**0.023**	0.941	0.641
ETR-LC	**0.012**	**0.007**	**<0.001**	0.626	**0.023**	0.941	0.642
F_v/F_m	**0.007**	0.844	**<0.001**	0.445	**0.003**	0.991	0.816
q_L	0.577	**<0.001**	**<0.001**	0.648	0.375	**0.012**	0.540
NPQ	0.097	0.113	**<0.001**	0.148	**0.020**	0.360	0.745
NDVI	**0.017**	0.813	**<0.001**	0.132	**0.002**	0.081	0.080
LRWC	0.123	0.739	**<0.001**	0.422	0.795	0.226	0.984
aTBioDW	0.504	**0.022**	**<0.001**	0.051	0.217	0.410	0.051
SLA	0.201	0.776	**<0.001**	0.272	0.573	**0.004**	0.937
aSpkN	**<0.001**	**<0.001**	**<0.001**	**<0.001**	0.199	0.519	0.095
aTillN	0.453	**0.008**	**0.001**	0.755	0.399	0.938	0.317
mTBioDW	0.810	**0.004**	**<0.001**	0.928	**0.022**	0.947	0.326
mGrainYield	**0.036**	0.327	**<0.001**	0.641	0.922	**0.030**	0.708
HI	**0.050**	0.696	**<0.001**	0.938	0.467	0.084	0.867
TGW	0.307	0.826	**<0.001**	0.509	0.894	**0.009**	0.330
mSpkN	**0.007**	0.589	**<0.001**	0.217	0.379	0.992	0.971
mTillN	0.391	0.768	**0.007**	0.577	0.692	0.896	0.772
mSpkDW	**0.014**	**0.025**	**<0.001**	0.365	0.544	0.140	0.390
mTillDW	0.052	0.083	**<0.001**	0.104	**0.006**	0.228	0.203
mLeafDW	**<0.001**	0.287	**<0.001**	0.897	**0.021**	0.167	0.278
