# Supplementary material for: Elevated CO2 Improves the Physiology but Not the Final Yield in Spring Wheat Genotypes Subjected to Heat and Drought Stress During Anthesis
Source: Front Plant Sci. 2022 Mar 7;13:824476. doi: 10.3389/fpls.2022.824476 (PMC8940247; doi:10.3389/fpls.2022.824476)
Supplement: Supplementary file 8 [file Table_3.pdf]

Supplementary Table S3. Three-way ANOVA of metabolites between genotype (G- SF29 or LM20), CO_2 levels (aCO_2 or eCO_2) and treatments (T- heat stress for 4 days (H4), heat stress for 7 days (H7) or combined drought and heat stress (D+H7)).

Type	Metabolite		G	CO_2	T	G × CO_2	G × T	CO_2× T	G × CO_2 × T
Sugars (nmol mg^{-1} DW)	FRUC	Fructose	**<0.001**	0.883	**<0.001**	**<0.001**	0.951	**<0.001**	**<0.001**
	GLUC	Glucose	**<0.001**	0.053	**<0.001**	**<0.001**	0.741	**<0.001**	**<0.001**
	SUC	Sucrose			**0.002**				
Organic acids (pmol mg^{-1} DW)	Cl	Chloride	0.544	**0.004**	**0.002**	0.118	0.065	0.235	**0.041**
	Citr	Citrate	0.264	0.488	**<0.001**	0.073	**0.009**	0.935	0.183
	Ma	Malate	**0.038**	**0.003**	**<0.001**	0.548	0.738	0.364	**0.040**
	NO_3	Nitrate	**0.001**	**0.042**	**<0.001**	0.286	**<0.001**	0.109	**0.034**
	PO	Phosphate	**<0.001**	0.057	**<0.001**	0.742	0.302	0.126	**0.003**
	SO	Sulfate	**<0.001**	0.792	**<0.001**	**0.008**	0.337	**<0.001**	**<0.001**
Free phenolics (pmol mg^{-1} DW)	4-HBA	4-Hydroxzbenzoic a.	0.305	**0.012**	**0.004**	0.839	0.503	0.653	0.582
	CHLA	Chlorogenic a.	**<0.001**	**0.013**	**<0.001**	**<0.001**	**0.015**	**0.034**	**0.002**
	FA	Ferulic a.	**<0.001**	**<0.001**	**<0.001**	**<0.001**	**<0.001**	**<0.001**	**<0.001**
	pCA	p-Coumaric a.	0.215	0.351	0.078	0.781	0.102	0.201	**0.035**
	SaA	Salicylic a.	0.082	**0.015**	**<0.001**	0.898	**0.004**	**0.021**	**<0.001**
	SaAG	Salicylic acid glucoside	**<0.001**	**<0.001**	**<0.001**	0.772	0.127	0.111	**0.002**
Free amino acids (pmol mg^{-1} DW)	AAA	2-Aminoadipic a.	**<0.001**	0.343	**<0.001**	0.711	0.323	0.720	0.267
	AcGlu	Acetyl glutamate	0.328	**0.037**	**<0.001**	0.227	**0.008**	**0.031**	0.619
	AcOrn	Acetyl ornithine	0.570	**0.050**	**<0.001**	**0.006**	**0.002**	0.143	0.240
	Ala	Alanine	**<0.001**	0.650	**<0.001**	0.258	0.321	0.051	**<0.001**
	Arg	Arginine	**<0.001**	0.062	**<0.001**	0.955	0.594	0.626	0.063
	Asn	Asparagine	**0.008**	**0.010**	**<0.001**	0.147	**<0.001**	0.068	0.053
	Asp	Asparagic a.	0.949	**0.005**	**<0.001**	0.307	**<0.001**	**<0.001**	**<0.001**
	BABA	β-Aminobutyric a.	**0.012**	0.378	**0.011**	0.322	0.129	0.339	0.856
	βAla	β-Alanine	0.692	0.080	**<0.001**	0.132	**0.035**	0.294	0.190
	Cit	Citruline	0.224	0.084	**<0.001**	**0.004**	0.212	0.572	0.503
	Cyst	Cysteine	0.061	**0.002**	**<0.001**	**0.005**	0.847	**0.002**	**0.014**
	GABA	γ-Aminobutyric a.	**<0.001**	0.062	**<0.001**	0.502	0.237	**0.002**	**<0.001**
	Gln	Glutamine	**0.028**	**0.001**	**<0.001**	0.093	0.178	0.175	0.160

	Glu	Glutamic a.	**<0.001**	**0.041**	**0.001**	0.904	0.730	**0.030**	0.770
	His	Histidine	0.984	**0.007**	**<0.001**	0.078	**<0.001**	**0.047**	0.145
	HomoArg	Homoarginine	0.289	**<0.001**	**<0.001**	**0.045**	**<0.001**	0.056	**0.002**
	Lys	Lysine	0.518	0.163	**<0.001**	**0.005**	**<0.001**	**<0.001**	0.068
	Met	Methionine	**<0.001**	**0.012**	**<0.001**	0.163	0.378	**0.037**	**0.021**
	Orn	Ornithine	0.814	**<0.001**	**0.016**	**<0.001**	**0.033**	0.766	0.873
	Phe	Phenylalanine	0.178	**0.044**	**<0.001**	0.204	0.286	0.092	**<0.001**
	Pro	Proline	0.185	**0.011**	**<0.001**	0.892	0.381	**0.008**	**0.002**
	Ser	Serine	0.162	0.054	**0.010**	0.147	0.151	0.764	0.633
	Thr	Threonine	0.159	0.077	**<0.001**	0.100	0.436	0.147	0.255
	Trp	Tryptophane	**0.005**	0.307	**<0.001**	**0.008**	0.292	0.185	0.106
	Tyr	Tyrosine	**0.022**	0.841	**<0.001**	0.512	0.087	0.126	0.069
	Val	Valine	0.143	0.704	**<0.001**	**0.041**	0.295	0.208	**0.029**
Free polyamines (pmol mg^{-1} DW)	Agm	Agmatine	**0.002**	0.356	**<0.001**	0.471	**0.004**	0.569	**0.022**
	Cad	Cadaverine	0.660	0.184	**<0.001**	0.267	0.962	0.975	0.142
	Dap	1,3-Diaminopropane	0.593	**<0.001**	**<0.001**	0.212	**0.008**	**0.003**	**<0.001**
	Hist	Histamine	0.762	0.198	**<0.001**	**0.011**	0.504	0.525	0.921
	NorSpm	Norspermidine	0.437	0.109	0.313	**0.021**	0.087	0.665	**0.005**
	Put	Putrescine	**<0.001**	**<0.001**	**<0.001**	**<0.001**	**0.007**	0.264	0.327
	Spd	Spermidine	**<0.001**	0.305	**<0.001**	**<0.001**	**0.008**	**0.007**	**0.005**
	Spm	Spermine	**<0.001**	**0.019**	**<0.001**	**<0.001**	0.728	0.396	0.078
